# A pretrained transformer model for decoding individual glucose dynamics from continuous glucose monitoring data

**DOI:** 10.1093/nsr/nwaf039

**Published:** 2025-02-08

**Authors:** Yurun Lu, Dan Liu, Zhongming Liang, Rui Liu, Pei Chen, Yitong Liu, Jiachen Li, Zhanying Feng, Lei M Li, Bin Sheng, Weiping Jia, Luonan Chen, Huating Li, Yong Wang

**Affiliations:** Center for Excellence in Mathematical Sciences, National Center for Mathematics and Interdisciplinary Sciences, Hua Loo-Keng Center for Mathematical Sciences, Key Laboratory of Management, Decision and Information System, Academy of Mathematics and Systems Science, Chinese Academy of Sciences, Beijing 100190, China; School of Mathematics, University of Chinese Academy of Sciences, Chinese Academy of Sciences, Beijing 100049, China; Department of Endocrinology and Metabolism, Shanghai Sixth People's Hospital Affiliated to Shanghai Jiao Tong University School of Medicine, Shanghai Diabetes Institute, Shanghai Clinical Center for Diabetes, Shanghai Key Laboratory of Diabetes Mellitus, Shanghai 200233, China; Key Laboratory of Systems Health Science of Zhejiang Province, School of Life Science, Hangzhou Institute for Advanced Study, University of Chinese Academy of Sciences, Hangzhou 310024, China; BGI-Research, Hangzhou 310030, China; School of Mathematics, South China University of Technology, Guangzhou 510640, China; School of Mathematics, South China University of Technology, Guangzhou 510640, China; Center for Excellence in Mathematical Sciences, National Center for Mathematics and Interdisciplinary Sciences, Hua Loo-Keng Center for Mathematical Sciences, Key Laboratory of Management, Decision and Information System, Academy of Mathematics and Systems Science, Chinese Academy of Sciences, Beijing 100190, China; School of Mathematics, University of Chinese Academy of Sciences, Chinese Academy of Sciences, Beijing 100049, China; Center for Excellence in Mathematical Sciences, National Center for Mathematics and Interdisciplinary Sciences, Hua Loo-Keng Center for Mathematical Sciences, Key Laboratory of Management, Decision and Information System, Academy of Mathematics and Systems Science, Chinese Academy of Sciences, Beijing 100190, China; School of Mathematics, University of Chinese Academy of Sciences, Chinese Academy of Sciences, Beijing 100049, China; Center for Excellence in Mathematical Sciences, National Center for Mathematics and Interdisciplinary Sciences, Hua Loo-Keng Center for Mathematical Sciences, Key Laboratory of Management, Decision and Information System, Academy of Mathematics and Systems Science, Chinese Academy of Sciences, Beijing 100190, China; Department of Statistics, Department of Biomedical Data Science, Bio-X Program, Stanford University, Stanford CA 94305, USA; Center for Excellence in Mathematical Sciences, National Center for Mathematics and Interdisciplinary Sciences, Hua Loo-Keng Center for Mathematical Sciences, Key Laboratory of Management, Decision and Information System, Academy of Mathematics and Systems Science, Chinese Academy of Sciences, Beijing 100190, China; Department of Computer Science and Engineering, Shanghai Jiao Tong University, Shanghai 200240, China; Department of Endocrinology and Metabolism, Shanghai Sixth People's Hospital Affiliated to Shanghai Jiao Tong University School of Medicine, Shanghai Diabetes Institute, Shanghai Clinical Center for Diabetes, Shanghai Key Laboratory of Diabetes Mellitus, Shanghai 200233, China; State Key Laboratory of Cell Biology, Center for Excellence in Molecular Cell Science, Shanghai Institute of Biochemistry and Cell Biology, Chinese Academy of Sciences, Shanghai 200031, China; Key Laboratory of Systems Health Science of Zhejiang Province, School of Life Science, Hangzhou Institute for Advanced Study, University of Chinese Academy of Sciences, Hangzhou 310024, China; Guangdong Institute of Intelligence Science and Technology, Zhuhai 519031, China; Pazhou Laboratory (Huangpu), Guangzhou 510555, China; Department of Endocrinology and Metabolism, Shanghai Sixth People's Hospital Affiliated to Shanghai Jiao Tong University School of Medicine, Shanghai Diabetes Institute, Shanghai Clinical Center for Diabetes, Shanghai Key Laboratory of Diabetes Mellitus, Shanghai 200233, China; Center for Excellence in Mathematical Sciences, National Center for Mathematics and Interdisciplinary Sciences, Hua Loo-Keng Center for Mathematical Sciences, Key Laboratory of Management, Decision and Information System, Academy of Mathematics and Systems Science, Chinese Academy of Sciences, Beijing 100190, China; School of Mathematics, University of Chinese Academy of Sciences, Chinese Academy of Sciences, Beijing 100049, China; Key Laboratory of Systems Health Science of Zhejiang Province, School of Life Science, Hangzhou Institute for Advanced Study, University of Chinese Academy of Sciences, Hangzhou 310024, China

**Keywords:** pretrained model, diabetes, continuous glucose monitoring, glucose dynamics

## Abstract

Continuous glucose monitoring (CGM) technology has grown rapidly to track real-time blood glucose levels and trends with improved sensor accuracy. The ease of use and wide availability of CGM will facilitate safe and effective decision making for diabetes management. Here, we developed an attention-based deep learning model, CGMformer, pretrained on a well-controlled and diverse corpus of CGM data to represent individual's intrinsic metabolic state and enable clinical applications. During pretraining, CGMformer encodes glucose dynamics including glucose level, fluctuation, hyperglycemia, and hypoglycemia into latent space with self-supervised learning. It shows generalizability in imputing glucose value across five external datasets with different populations and metabolic states (MAE = 3.7 mg/dL). We then fine-tuned CGMformer towards a diverse panel of downstream tasks in the screening of diabetes and its complications using task-specific data, which demonstrated a consistently boosted predictive accuracy over direct fine-tuning on a single task (AUROC = 0.914 for type 2 diabetes (T2D) screening and 0.741 for complication screening). By learning an intrinsic representation of an individual's glucose dynamics, CGMformer classifies non-diabetic individuals into six clusters with elevated T2D risks, and identifies a specific cluster with lean body-shape but high risk of glucose metabolism disorders, which is overlooked by traditional glucose measurements. Furthermore, CGMformer achieves high accuracy in predicting an individual's postprandial glucose response with dietary modelling (Pearson correlation coefficient = 0.763) and helps personalized dietary recommendations. Overall, CGMformer pretrains a transformer neural network architecture to learn an intrinsic representation by borrowing information from a large amount of daily glucose profiles, and demonstrates predictive capabilities fine-tuned towards a broad range of downstream applications, holding promise for the early warning of T2D and recommendations for lifestyle modification in diabetes management.

## Teaser

This article presents a pretrained transformer model for decoding individual glucose dynamics from continuous glucose monitoring data for diabetes screening, subtyping, risk stratification, and personalized dietary recommendations.

## INTRODUCTION

Type 2 diabetes (T2D) is a chronic disorder in glucose metabolism, which is characterized by β-cell dysfunction and insulin resistance [1,2] and driven by both genetic and environmental factors [3–5]. Current diagnosis of abnormal glucose metabolism relies on single-time-point static measurement or on average measures of overall glycemia, but ignores glucose dynamics [6], making it difficult to fully represent an individual's metabolic state and achieve a clear-cut diagnosis and classification of T2D. Moreover, the pathophysiological abnormalities, clinical manifestation, risk of complications, and response to therapeutic intervention in T2D patients may vary greatly among individuals [7–9].

Continuous glucose monitoring (CGM) systems furnish comprehensive and real-time data on glucose levels, enabling the detection of fluctuations and trends in blood glucose levels throughout the entire day and night [10]. Meanwhile, CGM helps individuals at risk of glucose dysregulation make informed decisions about food choices, exercise, and other aspects of diabetes management by knowing about daily glycemic patterns and fluctuations [11]. It's crucial to fully capture the glucose dynamics from CGM data to reap its maximum benefit in the research and clinical application of diabetes.

Recently, the advent of the self-attention mechanism has further captured large input spaces, learnt which elements are most important to focus on in each context, generated context-aware models, and boosted predictions in a wide range of applications [12,13]. Glucose dynamics are recorded as time series data in a highly context-dependent way. There are vast differences between individuals due to many factors such as weight, age, changes during pregnancy, diet or exercise. Attention-based transformer models hold promise to context-specific modelling of glucose dynamics from long-term continuous time series measurement. In addition, the concept of a foundation model has revolutionized fields such as natural language understanding for example BERT [14], GPT [15–17], PaLM [18,19], and LlaMA [20] and computer vision such as DALL-E [21,22], Flamingo [23], RETFound [24], DeepDR [25], DeepDR Plus [26]. It leverages deep learning transformer models pretrained on large-scale general datasets and achieves remarkable performance by fine-tuning towards a vast array of downstream tasks with limited task-specific data [14,27]. A foundation model could acquire broad information during the large-scale pretraining phase and fill the critical gaps for traditional methods in existing research for the usage of large volumes of high-quality labels. It could transfer knowledge to a multitude of downstream new tasks by overcoming the difficulty of yielding meaningful predictions by training a new model from scratch for each isolated task.

Here, we propose CGMformer, an attention-based transformer model pretrained on a diverse corpus of CGM data to capture an individual's glucose dynamics and enable clinical applications. We collected the Nationwide Multicenter CGM dataset for the pretraining phase with a total of 1917 days glucose measurements from 964 participants in China with matched comprehensive clinical information. We pretrained CGMformer using a self-supervised masked learning objective to capture the individual's glucose dynamics into the embedded vectors as an intrinsic representation, demonstrate predictive capability across five multiethnic datasets with different populations and metabolic states, and enable diverse clinical applications by fine-tuning towards a diverse panel of downstream tasks including screening of diabetes and complications, non-diabetes subtyping, and dietary recommendations. The intrinsic representation of glucose dynamics and performance improvement by pretraining is made robust by using large-scale unlabeled National Real-World CGM data with improved sensor accuracy and a glucose measurement from 58 847 users for totally 1 310 548 days. Overall, CGMformer represents a pretrained deep learning model which provides insights to an individual's overall glucose dynamics in fasting glucose homeostasis and postprandial glucose adaptation and has great potential to assist screening, subtyping, and treatment.

## RESULTS

### CGMformer architecture and pretraining

**Figure 1.**
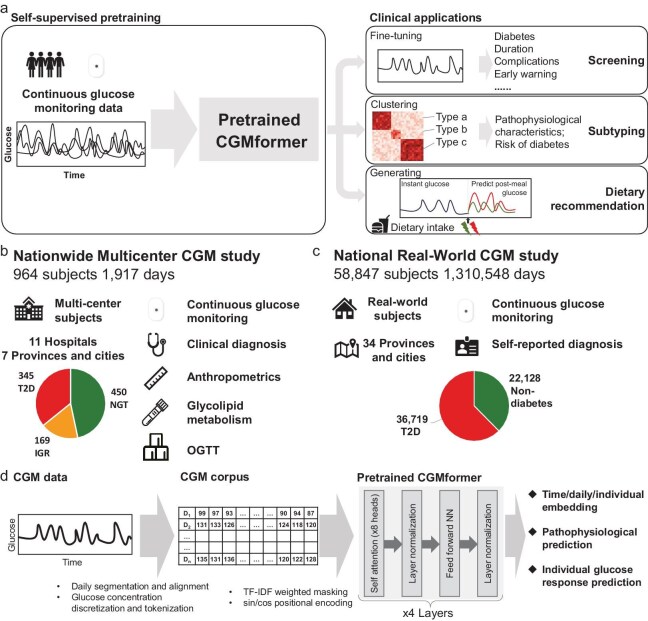
CGMformer architecture and pretraining. (a) Schematic of CGMformer. CGMformer is first self-supervised pretrained on CGM data to gain fundamental knowledge of the glucose dynamics, and then applied to a multitude of downstream clinical applications. The extractable contextual time point and individual embeddings can be used as an intrinsic representation for daily glucose profiles in clinical applications including screening, subtyping, and postprandial glucose prediction and dietary suggestion. (b) Overview of the Nationwide Multicenter CGM study. A total of 964 Chinese subjects were enrolled from 11 academic hospitals in China between 2007 and 2009, including 450 NGTs (normal glucose tolerance), 169 IGRs (impaired glucose regulation), and 345 T2Ds (type 2 diabetes). Participants recruited to the study were connected to a CGM system for three consecutive days. Meanwhile, comprehensive clinical information including anthropometrics and laboratory tests were taken for the study participants. (c) Overview of the National Real-world CGM study. CGM data from 58 847 Chinese subjects were collected in 2022 with improved sensor accuracy. (d) Pretrained CGMformer architecture. CGM records are first split by day and then tokenized to CGM corpus according to glucose records. The CGM corpus is then processed through four layers of transformer encoder units with eight attention heads per layer. Extractable output includes contextual time point and individual embeddings, contextual attention weights, and contextual predictions.

We develop CGMformer as an attention-based, context aware deep learning model pretrained on a large-scale and diverse corpus of CGM data to capture individual glucose dynamics and enable clinical applications [27]. CGMformer takes daily CGM glucose profiles as input and utilizes the recent advent of self-attention mechanism to gain fundamental knowledge in glucose dynamics across individuals and within individuals. With the extractable contextual individual and time point embeddings integrating with an individual's clinical or lifestyle information, CGMformer is able to help in diabetes screening, non-diabetes subtyping, and dietary recommendations (Fig. 1a, Methods).

Specifically, we first collected two large-scale CGM datasets, Nationwide Multicenter CGM study and National Real-World CGM data. In the Nationwide Multicenter CGM study, we recruited 964 Chinese participants from 11 hospitals of 7 provinces or cities directly under the Central Government in China, in 2007–2009 [28–30]. This dataset covers diverse glucose management states including 450 individuals with clinically diagnosed normal glucose tolerance (NGT), 169 with impaired glucose regulation (IGR), and 345 with T2D, with non-biased age distribution and balanced gender ratio (Fig. 1b, Note S2, Table S1, Fig. S1a–b). Participants received comprehensive laboratory tests including anthropometrics, glycolipid metabolism, and oral glucose tolerance test (OGTT) (Fig. 1b, Note S2, Table S1). In the National Real-World CGM data, we collected CGM records from 58 847 users in 2022 with self-reported metabolic states, including 36 719 T2Ds and 22 128 non-diabetes (Fig. 1c).

Each CGM records were segmented into single-day time series. We excluded the first and last uncompleted days of the CGM records and retained the other days with complete full-day CGM records, resulting in 1917 days CGM records for the Nationwide Multicenter study, and 1 310 548 days glucose records for the National Real-World CGM study (Fig. S2a–c, Note S2). The single day CGM records were then tokenized to assemble a corpus for pretraining. Specifically, the glucose values at each time point were discretized into 260 distinct glucose levels naturally by mg/dL unit (from 40 mg/dL to 300 mg/dL) to mimic a glucose-level sequence with length 288. The discrete tokenization approach offers a flexible and robust model that effectively captures the dynamic patterns and provides tolerance to the CGM measurement error (Fig. 1d, Methods). We further processed the assembled corpus through four transformer encoder units, each comprising a self-attention layer with eight self-attention heads and a feedforward neural network layer. Pretraining adopted a masked learning objective, a technique widely used in various domains to enhance the generalizability of foundational knowledge for diverse downstream fine-tuning objectives and applications (Fig. 1d, Methods).

During pretraining, 45%–60% tokens within CGM records were masked, and the model was trained by utilizing the context of the remaining unmasked glucose levels to predict the glucose level within each masked position. To glean more insights into abnormal glycemia, we gave tokens representing hyperglycemia and hypoglycemia higher mask weights by TF-IDF weighted masking [31]. We included sin/cos positional encoding in the transformer to ensure the continuity of time representation (Fig. 1d, Note S4). The CGMformer architecture is self-supervised and enables training on unlabeled data. This inherent strength in inclusivity allows for the incorporation of vast amounts of training data without the constraint of requiring large volumes of high-quality labels. We implemented recent advancements in distributed graphical processing unit (GPU) training to execute efficient pretraining on the large-scale dataset [32,33]. The mean vectors across all tokens in the final transformer layer were served as the embedding vector for the recorded day.

Subsequently, we trained CGMformer on the two collected divided datasets (Note S1, S7). We validated the model pretrained on the Nationwide Multicenter CGM data in internal and external datasets which involve different sample sizes, CGM devices, populations, and metabolic states with masking glucose prediction (Note S1). For a glucose sequence with 45%∼60% masked, CGMformer pretrained on the Nationwide Multicenter CGM data achieves higher accuracy in predicting hyperglycemia value (>180 mg/dL), hypoglycemia value (<70 mg/dL), and euglycemia value (70–180 mg/dL) across four external validation datasets, superior to traditional imputation methods including linear interpolation, KNN and other methods (Fig S3a–b). The results suggest that the pretrained CGMformer shows generalizability in predicting glucose values across five datasets with different populations and metabolic states. Moreover, with larger number of parameters (10 M) and training data volume, the model pretrained on National Real-World data shows improvement in pretraining glucose prediction accuracy on external datasets (Note S7–9, Fig S3c).

We conducted extensive ablation studies to evaluate the impact of pretraining loss, model architecture, embedding dimension, and masking strategy, with a focus on both pretraining token prediction accuracy and downstream screening performance (Note S9). Our results show that cross-entropy loss outperforms MAE loss in terms of downstream screening accuracy (Fig. S3d). We then compared the performance of the proposed Encoder-only architecture with another two popular pretraining architectures: *Encoder-Decoder* and *Decoder-only* (Note S9). The Encoder-only model outperforms the Decoder-only model and achieves comparable performance to the Encoder-Decoder model (Fig. S3e), with fewest parameters (Fig. S3f). Comparing different masking strategies including random masking proves the effectiveness of our self-adaptive TF-IDF masking strategy (Fig. S3g). When adjusting the latent space dimension for the model pretrained on the Nationwide Multicenter CGM data, we found that increasing the latent space dimension improved pretraining performance (Fig. S3h), but did not enhance the accuracy of downstream sample screening (Fig. S3i). In contrast, for the model pretrained on National Real-World data, performance improved as the model parameters increased from 0.8 million to 10 million, with significant gains observed when exceeding 6 million parameters (Fig. S3j), suggesting that larger datasets benefit from more complex models.

We further investigate the model's robustness to variations in input data through assessment of the accuracy of NGT/IGR/T2D screening. Prediction with down sampled data demonstrates that CGMformer maintains strong performance even when the input sequence is restricted to fewer measurements, longer measurement intervals, or when data is missing, ensuring its generative capability across different CGM device types (Fig. S3l, Note S9). When pretrained with fewer glucose level tokens, corresponding to lower resolution in glucose measurements, the model shows a decrease in accuracy by 8% with a 10 mg/dL unit (Fig. S3k), validating the effectiveness and robustness of our tokenization strategy. These results highlight the robustness of pretrained CGMformer in capturing glucose dynamics, demonstrating consistent generalizability and reliability across varying model architectures, parameter settings, CGM device types, and diverse datasets.

### CGMformer learns an intrinsic representation of individual glucose dynamics

**Figure 2.**
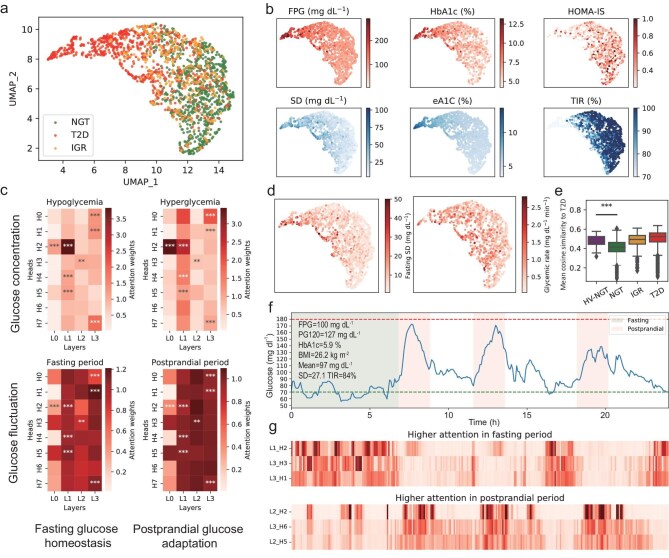
CGMformer adeptly captures individual glucose dynamics through attention. (a) UMAP visualizations of pretrained CGMformer's individual embeddings colored by NGT/IGR/T2D labels that capture a continuous trajectory from NGT to T2D. (b) UMAP visualizations of pretrained CGMformer individual embeddings with clinical- and CGM-derived measurements. Pretrained CGMformer individual embeddings align well with individual clinical- or CGM-derived measurements. (c) Pretrained CGMformer attention weights of token with abnormal glycemia including hyperglycemia (>180 mg/mL) and hypoglycemia (<70 mg/dL), as well as tokens within different fluctuation periods, including fasting and postprandial. (d) UMAP visualizations of pretrained CGMformer individual embeddings with mean fasting SD and mean glycemic rate. (e) Comparison of mean cosine similarity to T2Ds after being encoded by CGMformer among High-Variance NGT (HV-NGT), NGT, IGR and T2D. (f) CGM profile for an individual in HV-NGT with normal laboratory tests and CGM-derived metrics. (g) Higher attention weights to time points from different layers and heads in fasting period or postprandial period. Each row corresponds to an attention head denoted as Hn_Lm, where *n* signifies the head number and *m* denotes the layer number. The columns aligning with the CGM profile in *i* indicate the timepoints.

We next show that CGMformer autonomously learns the intricate dynamics of glucose value through the contextual attention weight as well as low-dimensional vector embedding in a latent space. We examined whether CGMformer provides individual embeddings preserving individual characteristics and effectively convey their clinical information. By visualizing the vectors encoded from pretrained CGMformer by projecting them into a two-dimensional space using Uniform Manifold Approximation and Projection (UMAP), we observed a discernible progression from NGT to IGR and onwards to T2D (Fig. 2a), indicating the consistency between diabetes state and glucose dynamics captured by CGMformer. Moreover, these vectors encapsulate HbA1c, FPG, and homeostasis model assessment for insulin sensitivity (HOMA-IS) [34] (Fig. 2b, Fig. S4a). Additionally, 48 CGM-derived metrics [35] correlated with diabetes state (Fig. S2a, Note S3) and laboratory tests (Fig. S2b) were calculated and clustered into three groups indicating glucose homeostasis, adaption, and in-range measure for glucose values (Fig. S2c). We selected three representative metrics in each group: standard deviation (SD), estimated A1C (eA1C) [36], and time in range (TIR) [37] showing their moderate consistency with CGMformer's embedded vectors (Fig. 2b, Fig. S4b). This robust representation of both clinical features and CGM characteristics in the latent space attest to the effectiveness of CGMformer in encapsulating diverse aspects of an individual's CGM profile, offering a comprehensive perspective for further analysis and clinical interpretation. Importantly, we noticed that our CGM embedded vector is better at recovering data for continuous disease progression while the laboratory tests (Fig. S4a) and single CGM-derived metrics (Fig. S4b) tend to show binary changes (Fig. 2b). Moreover, we tested if CGMformer provides stability to a sample representation across days. Since the National Real-World CGM data have on average over 12 days of glucose profiles from one individual, we tested the similarity of the embeddings between days from the same or a different individual. We observed that the inter-samples show significant lower similarity than intra-samples (*p* < 1 × 10^−8^, t-test, Fig. S4c), which suggest that CGMformer provides stable sample representation for an individual's metabolic state across days.

We then analyzed the extractable contextual attention weights of CGMformer for each head concerning specific glucose levels or time points to gain insights into how CGMformer captures individual glucose dynamic characteristics. Heatmaps were used to examine those attention weights in various contexts with regards to glucose concentration and fluctuation (Fig. 2c) [14,27]. Notably, abnormal glucose levels, encompassing hyperglycemia (>180 mg/dL) and hypoglycemia (<70 mg/dL), are prominently captured by the attention weights of the first two layers. In contrast, the last two layers demonstrate a heightened focus on specific times, such as the fasting or postprandial periods (Fig. 2c) [37]. Clearly distinct layers of CGMformer capture complementary information and the pretraining seems to recognize fasting and postprandial periods, identify meal timing, anticipate the effect of initial meals on subsequent glucose levels, and understand of the notion of diurnal insulin sensitivity. This observation supports that CGMformer exhibits a robust capability to capture both static homeostasis (fasting period) and the dynamic adaption to high glucose (postprandial period). Our layered architecture ensures a comprehensive and nuanced understanding of an individual's glucose profile, further emphasizing the versatility and efficacy of CGMformer in capturing diverse aspects of glucose dynamics.

CGMformer takes advantage of the self-attention mechanism and is able to capture the long-term association. Recent study shows that self-attention, which itself is the core novelty of transformers, entails a clustering effect [38]. As shown in Fig S4d–e, the learned glucose token encoded embeddings show both continuous glucose concentration and association among low and high glucose concentrations, demonstrating its ability to capture local and global patterns in CGM data. We conducted auto-regression with order selection for analyzing the complexity of glucose dynamics (Note S6), and observed that >50% of samples requires an average of >3-hour prediction order (Fig S4f), which indicates the common long-term association in glucose dynamics. Following the observation, we analyzed the encoder for glucose tokens before the first layer and indicate the long-term association between hypoglycemia and hyperglycemia revealed by CGMformer (Fig S4d and e). Moreover, CGMformer obtains additional metabolic insights for the glucoses beyond glucose concentration via quantization and self-supervised learning. For example, hyperglycemia overlaps with hypoglycemia at the second dimension in UMAP, which may indicate homeostasis deviation for the corresponding metabolic state.

CGMformer effectively captures glucose dynamics from CGM data, which are often overlooked by traditional glucose measurements that rely on single-time-point measurements or average values. For example, we focused on glucose variation during fasting periods and postprandial glycemic rates (Fig. 2d), which was proven to be highly associated with diabetes risk and outcome [39,40]. Among NGT participants, we identified a subgroup, termed High-Variance NGT (HV-NGT), characterized by relatively higher fasting period standard deviation and postprandial glycemic rates, resulting in a higher overall standard deviation compared to other NGTs (Fig. S5a and b). Despite these differences, traditional laboratory tests, including FPG, HbA1c, and post-meal 120-minute glucose (PG120), did not show significant differences between HV-NGTs and other NGTs (Fig. S5c). Notably, CGMformer's embedded vectors for HV-NGTs exhibited significant similarity to T2D profiles compared to other NGTs (t-test, *p*-value < 2.92 × 10^−13^, Fig. 2e, S5d). These findings suggest that CGMformer can identify a subset of NGT individuals with potential impaired glucose regulation. Taking the CGM profile from one of the HV-NGTs, *Shanghai_NGT_A183*, as an example, this participant shows normal FPG, PG120, and HbA1c levels (Fig. 2f), but large glycemic fluctuation during fasting and postprandial periods, which could be captured by the multi-head self-attention mechanism of CGMformer (Fig. 2g). A further two examples also show that the attention weights can learn the variability during fasting periods and low variability in the afternoon (Fig. S5e and f).

### CGMformer with fine-tuning assists clinical screening and prediction

**Figure 3.**
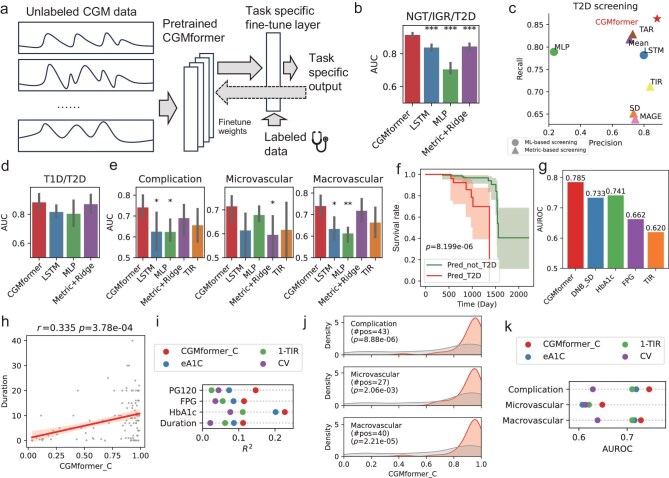
Fine-tuning CGMformer with labeled data assists screening and prediction. (a) Architecture for CGMformer fine-tuning towards a specific screening task. (b) Area under receiver operating characteristic curve (AUROC) of CGMformer fine-tuned to screening NGT/IGR/T2D from CGM data, compared to alternative methods. (c) Precision and recall score of CGMformer fine-tuned to identify T2D from ND, compared to alternative methods. (d) AUROC of CGMformer fine-tuned to identify T1D/T2D from CGM data using independent data, compared to alternative methods. (e) AUROC of CGMformer fine-tuned to identify complications and macro- or microvascular from CGM data using independent data, compared to alternative methods. (f) Survival curve for fine-tuned CGMformer predicted T2D and NDs in an independent longitude cohort. (g) AUROC for predicting follow-up outcome with CGMformer, DNB_SD, HbA1c, and FPG. (h) Scatter plot for CGMformer_C with diabetes duration. CGMformer_C significantly correlates with disease duration. (i) Correlation of CGMformer_C with laboratory tests, compared to alternative CGM-derived metrics. (j) Kernel density estimation (KDE) plot for distribution of CGMformer_C in samples with or without complications and micro- or macrovascular. (k) AUROC in predicting complications with CGMformer_C, compared to alternative CGM-derived metrics.

We next tested whether the pretrained CGMformer could transfer learned glucose patterns towards diverse downstream tasks via fine-tuning. CGMformer encodes the characteristics of glucose dynamics into an intrinsic representation specific to the context of each individual. Through supervised fine-tuning, CGMformer incorporates annotation labels by adding a task-specific layer and fully fine-tuning all transformer layers and output heads (Fig. 3a, Methods, Note S10). This approach tailors the model for precise prediction tasks with a carefully designed task-specific fine-tuning layer. We perform subsequent evaluations aimed to gauge the effectiveness of CGMformer for a diverse range of downstream fine-tuning applications when confronted with a shortage of labeled data. We consistently observed that pretraining yields better results than direct fine-tuning on a single task.

#### Fine-tuning CGMformer with labeled data assists clinical screening

We first fine-tuned CGMformer towards the NGT/IGR/T2D screening and compared it with alternative methods. Accuracy and AUROC are used for the evaluation of model performance. CGMformer outperforms machine learning methods, such as long short-term memory (LSTM) [41] and multi-layer perceptron (MLP) [42], with an average accuracy of 0.771 and AUROC of 0.914 (one vs rest) for NGT/IGR/T2D three classes in 5-fold testing using CGM records as input (Fig. 3b). Particularly, it significantly outperforms the LSTM by 8% in terms of both AUROC and accuracy (*p* = 1.478 × 10^−4^ for AUROC), and demonstrates the advantage of keeping track of arbitrary long-term dependencies in the input sequences by self-attention. When we combine these 48 metrics by machine-learning methods including ridge regression, MLP, and SGD (Note S14), CGMformer outperforms those predictors with statistically significant AUROC increase (Fig. S6b). In addition, the pretrained CGMformer outperforms a model with the same architecture but without pretraining, demonstrating the significant improvement of pretraining on performance (Fig. S6c). When the training data for fine-tuning is reduced, CGMformer maintains strong performance, highlighting the advantages of pretraining to borrow information to enhance robustness (Fig. S6d). For the identification of T2D, CGMformer outperforms the single metrics derived from CGM records, including mean glucose (Mean), SD, Mean Amplitude of Glucose Excursions (MAGE), TIR, and Time Above Range (TAR) (Fig. 3c, Note S14), and also LSTM and MLP. Among individuals with T2D, CGMformer shows great performance in predicting elevated FPG, PG120, and HbA1c, with recall rates of 0.890–0.967 (Fig. S6e). We delved deeper into the alterations of the attention weights after fine-tuning in task-specific supervised training. We found that 17 out of the 32 transformer units exhibit higher attention weights on tokens or time periods associated with homeostasis states, including hypoglycemia tokens and the fasting phase (Fig. S6f–g). This observation aligns with the fact that individuals are primarily labeled based on their glucose measurements and HbA1c.

Based on CGM data of 125 patients with diabetes in the Zhao *et al.* dataset [43], we fine-tuned CGMformer to identify patients with T1D or T2D. CGMformer consistently outperforms baseline methods including state-of-the-art machine learning (LSTM [41], MLP, etc.) as well as combining metrics-based predictors in identifying individuals with T1D or T2D (Fig. 3d, Fig. S6b), with accuracy of >0.9 in 5-fold cross validation.

Considering that the glucose dynamics pattern in CGM may inform the duration of diabetes and indicate its impacts on macrovascular and microvascular factors [44], we further investigated the performance of CGMformer in predicting diabetic complications, including the total complications, macrovascular complications, and microvascular complications. CGMformer achieves an accuracy of 0.8 in predicting microvascular complications and an accuracy of 0.7 in macrovascular complications, superior to other machine-learning based methods and metrics-based predictors (*p* = 4.418 × 10^−2^ comparing with LSTM in diabetic complication screening, Fig. 3e, Fig. S6b). Moreover, CGMformer outperforms TIR in the comparing of AUROC.

The CGMformer pretrained on the National Real-World CGM data with much larger parameters showed improvement across all the screening tasks (Fig. S6h), suggesting independent pretraining datasets show robust fine-tuning results and larger pretraining datasets bring higher representation ability. Together, the above finding indicates that CGMformer demonstrates its capability in assisting diabetes and complications screening based on a pretrained model and limited labeled data.

#### The fine-tuned CGMformer provides explainable early warning for T2D risk

CGMformer can accurately discriminate low- and high-risk groups for incident T2D based solely on CGM data (*p*-value < 8.2 × 10^−6^, Kaplan–Meier test, Fig. 3f) in Colas's dataset, which provides the incidence of T2D every 6 months during the follow-up period (6–72 months, median 33 months). CGMformer demonstrates its ability to provide early warnings for T2D occurrence with a predictive window of ∼3 years. To confirm that CGMformer learned the glucose dynamics for early warning, we employed the third-party dynamic network biomarker (DNB) method [45–47] to correlate with our results (Note S16). The DNB's SD calculation indicated that subjects identified by CGMformer exhibited significantly higher variation in glucose dynamics, despite having relatively similar HbA1c levels (Fig. S6i, Note S16). Furthermore, specimens exhibiting elevated SD by DNB are, to some extent, classified as IGR in the fine-tuned CGMformer with non-diabetes follow-up outcomes. In contrast, CGMformer demonstrates a mitigation of such false positives. CGMformer reaches the highest AUROC when predicting incident T2D, superior to SD, HbA1c, and FPG (Fig. 3g). These findings underscore the potential of CGMformer to provide explainable and clinically relevant early warnings for diabetes by capturing intricate aspects of glucose dynamics.

#### CGMformer provides a quantitative index for the impairment of glucose regulation

We next tested the ability of CGMformer to quantify the impairment of individual glucose regulation from its CGM profile. Compared with the CGM-derived single metrics such as eA1C, CV, and TIR, CGMformer integrates the embedded vectors that encompass abundant glucose dynamic characteristics and NGT/IGR/T2D label information from the individual in a supervised manner. To simplify the embedded vectors into low dimension, we proposed a multi-task deep learning–based framework to extract a single index CGMformer_C, to represent a simple yet more comprehensive understanding of the process of glucose regulatory impairment (Fig. S7a, Note S15). By integrating the clinical screening and measurements which indicate a comprehensive hemostasis metabolism status with the CGM embedding vector which provides dynamic characteristics, CGMformer_C is able to estimate the impairment of glucose regulation from CGM data. Intuitively, higher CGMformer_C indicates a more severe dysfunction of individual glucose regulation. CGMformer_C demonstrates its comprehensive ability to elucidate the state of glucose regulation and its correlation with clinical diagnosis and measurements from the Nationwide Multicenter CGM study (Fig. S7b–c). On Zhao's independent dataset, CGMformer_C exhibits a significant correlation with the duration of diabetes (Fig. 3h) as well as other laboratory tests (Fig. S7d), which offers an exciting opportunity to use the CGM profile to predict T2D duration since longer durations and poorer glucose regulation indicate a higher risk of complications. Moreover, CGMformer_C outperforms existing metrics including TIR, eA1C, and CV in correlating with diverse laboratory tests (Fig. 3i). CGMformer_C also shows a positive correlation with the risk of complications (Fig. 3j) and achieved the highest AUROC in predicting complications compared to other CGM-derived metrics (Fig. 3k). The results indicate that CGMformer_C achieves superior performance in predicting clinical characteristics and diabetic complications than other CGM-derived metrics, providing a valuable tool to evaluate glycemic status in individuals.

### CGMformer subtyping non-diabetes subtypes with diverse metabolic characteristics

We next show that CGMformer can enhance the early identification of abnormal glycemic status in individuals with NGT and prediabetes based on CGM data, preceding the onset of clinical manifestation of T2D. As OGTT might fail to capture the complete dynamics of glucose adaptation due to the static time points [48–50], CGMformer might provide subtypes that are more closely related to diabetes risk and offers the possibility for refining diabetes management.

**Figure 4.**
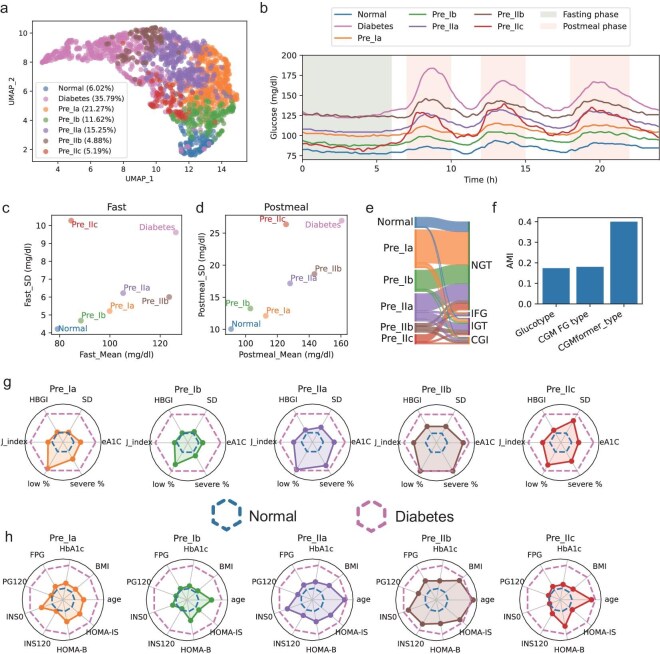
CGMformer enables detailed and comprehensive subtyping for non-diabetes. (a) UMAP visualizations for individuals in CGMformer_type. Non-diabetic individuals were classified into one normal subtype and five pre-diabetic subtypes. (b) Median CGM profile for subtypes, offering insights into the unique glucose dynamics of different subtypes. (c, d) Schematic representations of mean and variation during the fasting and postprandial phases for each subtype. (e) Correspondence between CGMformer_type and OGTT labels. (f) AMI for CGMformer_type and Glucotype, CGM FG type with OGTT labels. (g, h) Characteristics extracted from both CGM and laboratory tests for each subtype. HBGI: high blood glucose index. low%: fraction of time with low glucose variability, calculated from glucotype; severe %: fraction of time with severe glucose variability, calculated from glucotype.

We hierarchically clustered the NGT and IGR samples with the embedded vectors generated from pretrained CGMformer. We identified six clusters showing distinctive median CGM patterns in UMAP visualization (Fig. 4a, Methods). Clusters are named according to the percentage of NGTs and T2Ds in the group and roughly there are three clusters identified as Normal, Pre_I, and Pre_II, with an increasing ratio of IGRs. We further divided the Pre_I and Pre_II clusters to finally give one normal cluster and five prediabetes clusters, named as CGMformer_type (Fig. 4a).

We summarized the distribution of anthropometrics and pathophysiological characteristics in Table 1 within different time periods and compared the CGM-derived metrics (Fig. S8a) and laboratory tests (Fig. S8b) of different clusters (Table S2). The median profiles of CGMs from these subtypes, when juxtaposed with samples from individuals with diabetes, exhibit discernible patterns (Fig. 4b–d). Pre_IIb stands out with the highest glucose levels among non-diabetic individuals, closely resembling the diabetic group. During the fasting phase, Pre_IIb exhibits analogous glucose levels to those with diabetes but demonstrates superior glucose adaptation, which is evident in lower postprandial glucose levels and variability. Conversely, Pre_IIc manifests relatively lower fasting phase glucose levels but experiences substantial glucose fluctuation after meals (Fig. 4b–d). Normal, Pre_Ib, and Pre_Ia shows elevated average glucose concentration, but remains similar and with relatively low glucose variability. Whereas Pre_IIa, with a higher glucose concentration, shows significantly higher glucose variability when compared with Normal, Pre_Ib, and Pre_Ia.

**Table 1.** Characteristics of subtypes in CGMformer_type.

	Subtypes of non-diabetes stratified by CGMformer_type					
	Normal	Pre_Ia	Pre_Ib	Pre_IIa	Pre_IIb	Pre_IIc
***Key features***						
Obesity	Normal	Normal	Normal	Normal/overweight	Overweight/obesity	Normal
Insulin sensitivity	Good	Low	Average	Low	Very low	Average
Insulin secretion	Adequate	Adequate	Adequate	Adequate	Moderately low	Low
Mean glucose	Low	Moderate	Low	Moderate	High	Moderate
Glycemic variability	Low	Low	Low	Moderate	Moderate	High
Risk	Very low	Low risk	Low risk	Moderate risk *Age-related*	High risk *Obesity-related* *Insulin-resistant*	High risk *Beta-cell dysfunction* *Insulin-deficient*
***Recommended intervention***						
Person-centered care goals				Weight loss and maintenance, minimizing the progression of hyperglycemia, and attention to cardiovascular risk	Weight loss and maintenance, minimizing the progression of hyperglycemia, attention to cardiovascular risk, and intensive preventive approaches	Minimizing the progression of hyperglycemia, and attention to cardiovascular risk
Screening and assessment	Routine screening	Routine screening	Routine screening	Screening for diabetes or prediabetes	Screening for diabetes or prediabetes	Screening for diabetes or prediabetes
Weight management					Achieve and maintain a weight reduction of at least 7% of initial body weight	
Dietary administration	Healthy food-based dietary patterns	Healthy food-based dietary patterns	Healthy food-based dietary patterns	Healthy reduced-calorie diet	Healthy reduced-calorie, low-carbohydrate diet	Healthy food-based dietary patterns, low-carbohydrate diet
Exercise intervention	Moderate intensity physical activity	Moderate intensity physical activity	Moderate intensity physical activity	Moderate intensity physical activity	Moderate intensity physical activity	Moderate intensity physical activity
Pharmacologic Management					Metformin for adults at high risk of type 2 diabetes, especially those aged 25–59 years with BMI ≥35 kg/m^2^, higher fasting plasma glucose (≥6 mmol/L), and higher A1C (≥6.0%), and in individuals with prior gestational diabetes mellitus	

Notably, the majority of participants categorized as Normal, Pre_Ia, and Pre_Ib were diagnosed as NGT, while individuals in Pre_IIb were predominantly diagnosed with IGR or impaired glucose tolerance (IGT)/combined glucose tolerance (CGI) by OGTT [48] (Fig. 4e). We conducted further comparison with a glucose variation-based subtyping approach (glucotype) [6], which leverages CGM data and spectral clustering to subtype pre-diabetes based on glucose fluctuations, and a fasting glucose base subtyping (CGM FG type) [51]. CGMformer_type demonstrates high overall concordance with glucotype and CGM FG type, but provides a more granular insight into the intricate landscape of glucose dynamics (Fig. S9c and d). Moreover, CGMformer_type reaches the highest Adjusted Mutual Information score (AMI) with the OGTT diagnosis when compared with the other two subtyping methods (Fig. 4f). Those outperformances over the direct and individual CGM profile clustering results demonstrate the added value of pretraining. By borrowing information from a large and diverse cohort, CGMformer may reveal stable and intrinsic non-diabetes subtypes.

The CGM patterns of CGMformer_type support the fact that those participants show similar fluctuation levels but may be caused by different underlying mechanisms (Fig. 4g). We further compared the characteristics of different CGMformer_type (Fig. 4h). Consistent with the preceding findings, individuals in Pre_IIb exhibit poorer metabolic profiles, marked by advanced age, being overweight or obese, reduced insulin sensitivity, and hyperinsulinemia. Notably, participants classified as Pre_IIc show lower FPG and PG120 levels but displayed β-cell dysfunction and insulin deficiency, evident through lower fasting and postprandial serum insulin levels, indicating a role in the pathogenesis of diabetes. Pre_Ia and Pre_IIa both present with a slight insulin deficiency and relatively low insulin sensitivity. Pre_IIa, characterized by higher age, suggests potential age-related glucose regulatory changes. Pre_Ib mostly exhibits similar characteristics to the normal subtype. By clustering the samples encoded by model pretrained on the National Real-World CGM data, we observed consistent subtyping and corresponding CGM patterns (Fig. S8e). This suggests that the subtyping is robust across models from diverse pretrained data.

### CGMformer_type shows elevating onset and genetic risks for diabetes

We further validated the CGMformer_type on external datasets and assessed its onset and genetic risks given its potential useful biofeedback that could inspire non-diabetics to commence lifestyle changes. We validated our CGMformer_type of non-diabetes on two independent cohorts, the Colas dataset [52] (Note S1), which conducted a longitudinal follow-up study for the development of diabetes, and the CGMap, which collected CGM data from 7000 Israeli non-diabetic individuals [53] as a part of the Human Phenotype Project (HPP), to further characterize our CGMformer_type in non-diabetic populations with their matched comprehensive genomics and phenomics data.

**Figure 5.**
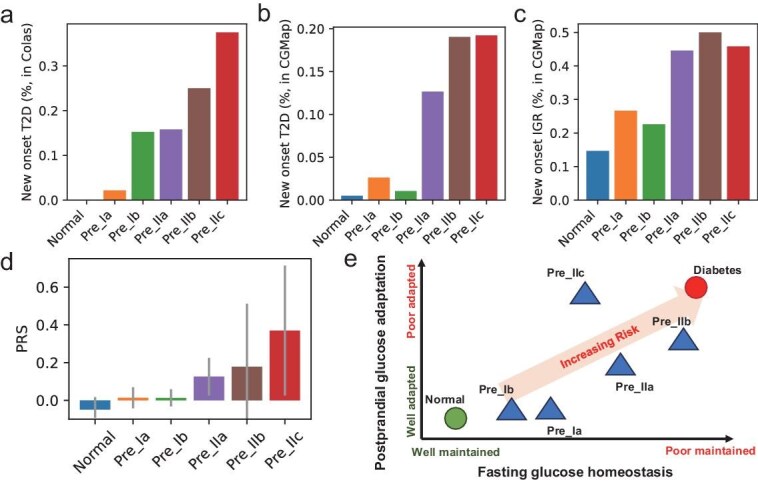
CGMformer_type shows elevated risk for diabetes. (a–c) Diabetes risk for each subtype, validated in new onset T2D from Colas's dataset (a); new onset T2Ds from CGMap (b); new onset IGR from CGMap (c). (d) Box plot for PRS of diabetes for samples with each CGMformer_type from CGMap. (e) Schematic representation of subtypes elucidates glucose regulatory dynamics, encompassing fasting glucose homeostasis and postprandial glucose adaptation.

In the Colas dataset, CGM records are encoded by the pretrained CGMformer and assigned to each subtype (see Methods). Participants categorized as Pre_IIb exhibited the highest propensity for developing diabetes, 37.5% (3 out of 8) progressed to diabetes, 25% (1 out of 4) in Pre_IIc developed diabetes, while individuals in other subtypes exhibited a lower incidence rate (Fig. 5a). This observation aligns with the preceding results and analysis. In CGMap, considering the limited accessibility, we trained a classifier based on the CGM-derived metrics of annotated reference data (our Multi-center CGM data) and its corresponding subtypes in order to annotate the samples from CGMap (Fig. S9a, Note S18) with CGMformer_type. The classifier takes the comprehensively derived statistics metrics from CGM data as input and outputs the corresponding subtype. We achieved >80% accuracy in predicting subtypes and this ensures the feasibility in transferring subtypes across different cohorts (Fig. S9b). We further validated the risk for new-onset IGR or T2D of each subtype in CGMap, according to criterion from their recent work [51]. HPP conducted a follow-up study on a subset of 4130 participants. Over a median follow-up of 910.5 days, 770 participants developed IGR and 83 participants developed T2D. The results showed that CGMformer_type identified individuals with high-risk of IGR and T2D, which is consistent with our previous results (Fig. 5b–c). Additionally, we observed that the samples in six subtypes from CGMap show similar clinical features to those in our Nationwide Multicenter CGM study (Table S3).

With the matched whole genome sequencing (WGS) and CGM data from CGMap, we further investigated the genetic risk of CGMformer_type. Taking the 1289 T2D risk SNPs from Ken *et al.* [54], we calculated the polygenetic risk score (PRS) of samples (Note S19). As shown in Fig. 5d, Pre_IIc showed the highest genetic risk for T2D, and Pre_IIb and Pre_IIa exhibited higher genetic risk than samples with Normal, Pre_Ia, or Pre_Ib. Utilizing the genetically heterogeneous groups revealed by the T2D SNP analysis of Ken *et al.* [54], group-specific PRS was calculated. The CGMformer_type showed diverse genetic features within different groups (Fig. S9c). Among them, Pre_IIc exhibited a high risk for T2D with insulin deficiency and significantly higher PRS for Beta cell-PI. Pre_IIb showed a high risk for obesity and higher PRS for lipodystrophy. Specifically, 21 SNPs which significantly associate with at least one subtype (Chi-squared test *p* < 0.005) are identified from the candidate SNPs (Fig. S9d), and the SNP in GPSM1 showed a significant and strong association with Pre_IIb.

Overall, we leveraged the matched follow-up outcome and genomic data with CGM data from two external datasets, revealed the onset genetic risk for T2D in each CGMformer_type, and assigned it with specific genetic features. CGMformer_type offers a comprehensive subtyping of non-diabetic individuals, which provides valuable insights into glycemic homeostasis and adaptation mechanisms and yields distinct profiles with variable diabetes risk. This could deepen our understanding of subtypes for non-diabetes captured by glucose dynamics in CGMformer and provide useful feedback for non-diabetics’ lifestyle adjustment under risks.

Based on these results, we can schematically represent the glucose characteristics of the subtypes from two perspectives: fasting glucose homeostasis and postprandial glucose adaptation (Fig. 5e). Individuals with normal subtype exhibit well-controlled homeostasis and effectively adapt to dietary intake, while those with diabetes show the opposite pattern. The Pre_Ia and Pre_Ib subtypes have slightly elevated mean glucose levels but still adapt well, demonstrating relatively normal insulin sensitivity and secretion. In contrast, the Pre_IIc subtype displays high glucose variability, indicating a significantly higher risk for developing diabetes.

### CGMformer predicts postprandial glucose and provides personalized dietary recommendations

CGMformer demonstrated its ability to capture an individual's glucose dynamics encompassing both homeostasis and adaptive responses to perturbations like meal intake. We utilize its power to predict personalized postprandial glycemic response to real-life meals and provide dietary recommendations. Lifestyle management, including dietary and exercise interventions, has proven effective in enhancing glucose control for individuals with diabetes [55–58]. Notably, dietary changes exert an immediate and highly correlated impact on postprandial glucose dynamics but individual response to the same meal is highly heterogeneous [59,60], which highlights the importance of personalized dietary intervention.

**Figure 6.**
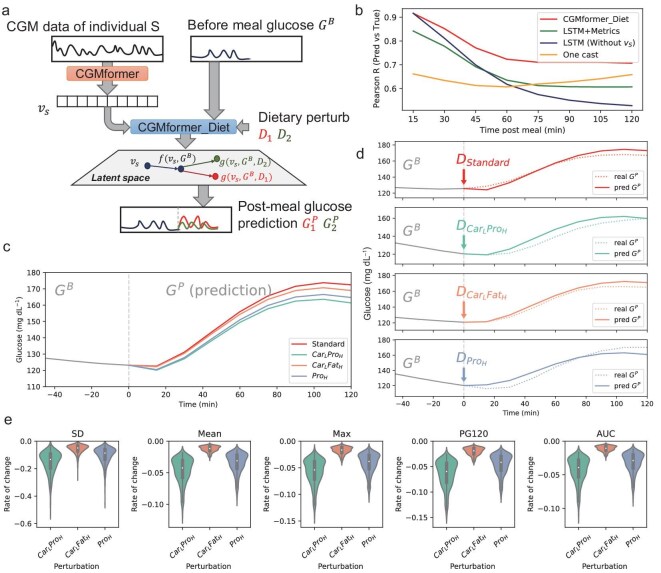
CGMformer_Diet predicts personalized postprandial glucose and suggests diet prescription. (a) Schematic of CGMformer_Diet. CGMformer_Diet generates predictions for postprandial glucose levels following dietary perturbations, leveraging individual embeddings encoded by CGMformer, before-meal glucose values, and meal intake information. (b) Correlation of predicted postprandial glucose with real glucose values from CGMformer_Diet, compared with baseline methods. (c) Predicted postprandial glucose after different meal intakes. (d) Comparison of CGMformer_Diet predicted postprandial glucose with real postprandial glucose for various dietary intakes. (e) Rate of change for metrics derived from postprandial glucose when perturbing meal intake from standard balanced meal.

We introduce CGMformer_Diet, a model built upon CGMformer, designed to predict postprandial glucose by integrating individual CGM records, real-time glucose data, and dietary information, including nutritional content (Fig. 6a, Fig. S10a, Methods). We combine the CGM data of individual, before meal glucose and dietary perturbation in the latent space and output the postprandial glucose prediction. The model was trained and tested in Zhao's dataset, aligning meal information with glucose dynamics and considering nutrition content such as calories, carbohydrates, proteins, fats, and dietary fiber.

CGMformer_Diet demonstrates accurate and robust postprandial glucose prediction with Pearson correlation coefficient and mean squared error with true glucose values in the whole 2-hour period (Fig. 6b, S10b). CGMformer_Diet outperforms the one-cast model which takes the same inputs but predicts the postprandial glucose directly with an MLP regression, which demonstrates the effectiveness of the auto-regression architecture. Moreover, it clearly surpasses the baseline model LSTM [41] with identical architecture but without the CGMformer encoded vectors as input. We further compare CGMformer_Diet with a model which substitutes the embedding from CGMformer with CGM metrics as individual features, and CGMformer_Diet also outperforms that. These outcomes highlight the significance of the vectors encoded by CGMformer during pretraining with unlabeled CGM data and they offer crucial information for understanding individual glucose dynamic characteristics and responses to perturbation.

We next devised an *in-silico* dietary perturbation experiment based on the predictive model by leveraging the predictive capability of CGMformer_Diet for individual postprandial glucose dynamics (Note S21). This requires designing simulated meals with fixed calories and adjusting the ratios of the three major nutrients—carbohydrates, protein, and fat. We chose four different simulated meals with different energy supply ratios of carbohydrates, protein, and fat, including a standard balanced meal and three adjusted meals (Note S21). These meals served as *in-silico* perturbations to dietary intake. The model provides various postprandial glucose predictions for different dietary scenarios (Fig. 6c), and the predictions aligned well with the grouped input meals based on energy supply ratios of nutrition, including carbohydrate, protein, and fat (Fig. 6d, S10c–d). This validation confirms the model's ability to predict postprandial glucose dynamics under different dietary conditions.

To offer recommendations based on dietary predictions and perturbations, we extract five metrics from postprandial glucose values, including the postprandial mean glucose (Mean), the postprandial 120 min glucose (PG120), postprandial max glucose (Max), SD, and AUROC. These metrics’ rate of change after perturbation were calculated to compare simulated diets with the standard diet. The results align with the consensus that decreasing carbohydrate intake is beneficial for controlling mean glucose levels and reducing glucose variation (Fig. 6e). Additionally, an increased protein ratio appears to be advantageous, while an excess of fat may contribute adversely to glucose dynamics. The results suggest that CGMformer holds potential for tailoring precise and effective dietary interventions for individuals with diabetes.

## DISCUSSION

Considering the occult and heterogeneous pathophysiology of diabetes [2], early detection and effective intervention based on an individual's full glucose dynamics are critical for the prevention and management of diabetes. In this study, we developed a context-aware deep-learning model, CGMformer, pretrained on CGM data to assist diabetes screening, non-diabetes subtyping, and dietary recommendation. Through self-supervised learning on large-scale unlabeled data, CGMformer gained a fundamental understanding of glucose dynamics, which improved the performance in assisting clinical screening of diabetes and subtyping non-diabetes. CGMformer_Diet is further put forward to provide precise prediction of an individual's postprandial glucose, and further *in-silico* perturbation of dietary intake indicated its potential to provide recommendations for lifestyle intervention. Our major contribution is both in creating an intrinsic representation for daily glucose profiles by pretraining and in covering diverse downstream tasks by transfer learning compared with existing dynamics, statistical and machine learning analysis [61–68]. These results demonstrated that CGMformer may serve as an adjunctive tool to promote the identification of high-risk individuals and personalized lifestyle intervention for diabetes management.

Unlike HbA1c and fasting glucose tests, which provide snapshots of glucose levels, CGM offers continuous monitoring, capturing variations throughout the day and night [10]. The duration of diabetes is closely associated with various manifestation and risk of complications [2,44]. As the disease progresses, T2D patients experience further deterioration of β-cell function and increased insulin resistance, leading to increased glycemic variability, and higher and prolonged postprandial glucose excursions [3,69,70]. In this study, CGMformer could provide more information of diabetes duration and exhibits great performance in the identification of diabetes and its complications. Furthermore, CGMformer_C, a synthesized index from CGMformer, demonstrated a significant correlation with diabetes duration, laboratory tests including PG120, FPG, and HbA1c, and the risk of complications, providing a valuable tool for assessing the state of glucose regulation in individuals. By detecting subtle changes in glucose dynamics and identifying glucose trends and patterns early, CGMformer can help prevent the progression of diabetes and its complications, reducing the overall disease burden. Currently, there are no CGM-based criteria for diabetes diagnosis, but international statements and consensus for application of CGM in diabetes management have been established [10,37,71,72]. Our work may have the potential to provide evidence on the efficacy and reliability of AI-driven CGM data in detecting diabetes-related glucose abnormalities. As CGM and AI technologies continue to advance, we foresee its potential integration into diagnostic protocols, offering a more dynamic and comprehensive approach to diabetes management.

CGMformer provides an attention-based deep learning model for subtyping of non-diabetic individuals based on CGM data alone, resulting in the identification of six distinct clusters characterized by diverse CGM patterns, clinical characteristics, and risk of diabetes. In our study, Pre_IIb was predominantly diagnosed with IGR or IGT/CGI and identified as the very high-risk subtype for diabetes and high propensity for new-onset diabetes. It showed a genetic risk for obesity, a higher PRS for lipodystrophy, and a significant association with GPSM1, which has been extensively studied for its impact on obesity, insulin resistance, and diabetes [73,74]. Previous studies have demonstrated a strong association between obesity, insulin resistance, and diabetes [75], highlighting insulin resistance as a probable underlying pathophysiology for Pre_IIb. Interestingly, individuals classified as Pre_IIc had relatively normal BMI and glucose levels, but showed significantly lower insulin secretion and higher glycemic variability. It also exhibited a high risk for T2D with insulin deficient and a significantly higher PRS for Beta cell-PI [76], which indicates the crucial role of β-cell dysfunction in the development of diabetes within this cluster. A study of the Hong Kong Diabetes Registry reported that patients with diabetes diagnosed before the age of 40 years had higher risks of all-cause death and cardiovascular–renal events than those diagnosed after that age, and >20% of people diagnosed with T2D before the age of 40 years were normal weight [77,78]. Some studies also found that lean patients with T2D had distinct clinical characteristics, gut microbiota, and risks of diabetic complications compared with obese patients with T2D [79–81]. Thus, Pre_IIc, with relatively normal weight and glucose levels, might be overlooked but require early detection and timely treatment. CGMformer could detect trends and anomalies in glucose levels that may not yet be apparent with traditional fasting glucose or HbA1c tests, and identify subtle glucose pattern abnormalities indicative of prediabetes or individuals with high T2D risk [82–84]. This early detection can facilitate timely intervention and potentially delay or prevent the onset of diabetes.

Integrating CGM data with AI technologies offer a holistic view of glucose dynamics, improving the understanding and management of diabetes beyond what current intermittent testing provides. T2DM is a very heterogeneous disease [80]. Some studies reported that ∼50% of patients with type 2 diabetes do not achieve adequate control [85], resulting in health and economic burden, micro- and macrovascular complications. This also underlines the many unmet needs and challenges people with diabetes have in the daily management of their condition. Evidence from clinical research has demonstrated that CGM devices can improve glycemic control for people with T2DM, as well as more-timely treatment intensification, lower risk of diabetes complications and hospital admissions [86–88]. However, the issue of real-time feedback and recommendation on dietary and exercise interventions in CGM systems still needs to be addressed. Previous studies reported accurate postprandial glycemic responses to simple, identical, and standard meals [59,60,89], but such meals are not representative of multicomponent meals in free-living conditions. In our study, CGMformer_Diet demonstrated accurate and robust postprandial glucose prediction based on various meals, suggesting that our model could offer crucial information for understanding individual glucose dynamic characteristics and responses to perturbation. Furthermore, through *in-silico* perturbations of dietary intake, the model demonstrated its potential to offer personalized suggestions for lifestyle modifications based on individualized glucose dynamics, without the need for prior meal records as a prerequisite for training. These findings support the fact that our model may be reliable and informative for developing personalized diet recommendations.

When pretrained on two diverse datasets, CGMformer demonstrates robust performance across various tasks, including glucose imputation, diabetes screening, and subtyping. The datasets offer complementary advantages for model development: the Nationwide Multicenter CGM data provides well-controlled collection conditions and comprehensive clinical information, while the National Real-World data offers a much larger volume of data with more accurate devices. However, the larger National Real-World CGM dataset requires a more complex model. This indicates that larger models are better equipped to handle diverse CGM data distributions and tasks, uncovering hidden patterns and providing deeper insights into glucose dynamics.

This study has several limitations. First, one-day CGM records were used for pretraining. Our results showed that vectors from the same individual exhibited significantly higher similarity in the latent space compared to those from different individuals, underscoring the stability of daily embeddings within the same individual. Considering that daily variations provide valuable insights into disease status, longer CGM records spanning several days will be utilized for pretraining in future work. Second, glucose dynamics are influenced by various factors, particularly lifestyle elements such as diet, sleep, exercise, and the use of drugs or external insulin. With sufficient records of these events, the model has the potential to provide more comprehensive sample representations and treatment recommendations. Third, the development of diabetes and its complications result from the interplay of genetic, phenotypic, and behavioral factors associated with chronic exposure to dysglycemia. Our study was focused on extracting individual glucose dynamics from CGM data to predict complications or assess glucose metabolism impairment solely from CGM data. Including these additional factors could further enhance the model's utility. Moreover, we pretrained CGMformer on two East Asian CGM datasets, which only included NGT/IGR/T2D samples. Although the pretrained model demonstrated generalizability across datasets from different races and disease statuses, pretraining on a more diverse dataset is worth exploring. Last, while preliminary results are promising, further clinical trials are necessary to validate the efficacy and safety of using AI-driven CGM data for diabetes management.

In conclusion, we developed the CGMformer model based on CGM data to capture glucose dynamics and enable clinical applications. By self-supervised pretraining on diverse CGM data, CGMformer learns intrinsic representations from glucose dynamics and enables versatile downstream applications. In the supervised scenario, CGMformer demonstrated its proficiency in clinical screening when fine-tuned with specific tasks. Additionally, there are currently no universally accepted diagnostic criteria of diabetes based on CGM data. We are optimistic that in the future, as AI and CGM systems evolve [90,91], AI-driven CGM data analysis will be an important tool for the screening and management of diabetes. For the unsupervised subtyping of non-diabetic individuals, CGMformer identified six distinct clusters characterized by diverse CGM patterns and clinical features. Our CGMformer_types may help people without diabetes better understand potential T2D risk and shape healthier lifestyle choices. Moreover, CGMformer could predict postprandial glucose responses, showcasing its ability to provide precise insights into postprandial glycemic patterns and personalized dietary guidance. Taken together, our CGMformer model has great potential to be an ancillary tool for clinical screening assistance, non-diabetes subtyping, and personalized dietary recommendations in diabetes management.

## METHODS

### CGMformer architecture and pretraining

#### CGMformer architecture

CGMformer is composed of four transformer encoder blocks, each composed of an eight-head self-attention layer and feed forward neural network layer. CGMformer takes 1-day CGM records $G \in {\mathbb{R}}^{288}$ as input, and outputs a latent embedding for the sequence ${v}_G \in {\mathbb{R}}^{128}$. For a sample with multi-days CGM records, the average embedding of each day serves as the representation of the sample.

#### CGM data preprocessing and glucose value tokenizing

The collected CGM data were first split into single-day segments from 00:00 to 23:59 based on the recorded timestamps. To minimize noise, records from the first and last days were excluded. Glucose values were then clipped to a physiological range of 40 to 300 mg/dL. During tokenization of the single-day CGM data, glucose values were discretized into 260 distinct glucose levels and ordered by time points to mimic a sentence structure. The missing measurements were imputed with <PAD> token. And a <CLS> token was added to the start of the sequence, which results in a sequence with a length of 289 for each daily CGM record.

#### CGMformer pretraining

Pretraining is achieved through self-supervised learning. Specifically, part of the token in the input sequence is masked and then input into the model, the masked tokens are predicted, and the cross-entropy loss is employed to optimize the model, which is defined as follows:


\begin{eqnarray*}
{\mathcal{L}}_{tokens} = {{\mathrm{\Sigma }}}_{j \in {\mathcal{T}}_{mask}}{y}_j\log q\left( j \right)/\left| {{\mathcal{T}}_{mask}} \right|,
\end{eqnarray*}

where ${\mathcal{T}}_{mask}$ denotes the set of masked tokens, ${y}_j$ denotes the true probability distribution of the token which is 1 at the true token *j* and 0 elsewise, and $q( j )$ denotes the predicted probability distribution for the token at the masked position. For the masking process, we propose a TF-IDF adaptive masking strategy with weighted masking for the glucose tokens (See Note S4).

### CGMformer fine-tuning

Fine-tuning of CGMformer was accomplished by initializing the model with the pretrained CGMformer weights and adding a final task-specific transformer layer. A one-layer neural network is then applied as the classification head following the task-specific transformer layer and transferred the token embeddings into the probability for each label. Part of the weights in the pretraining layers are frozen, and the rests and the task-specific layers are optimized to minimize the fine-tuning objective, which is task specific. Cross-entropy loss was employed as the label prediction loss, calculated as:


\begin{eqnarray*}
{L}_{{\mathrm{Pred}}} = - \sum\limits_{i = 1}^M {{z}_i\,\,{\mathrm{log}}\left( {{q}_i} \right)},
\end{eqnarray*}

where *M* is the number of CGM sequences, ${z}_i$ and ${q}_i$ indicate the ground-truth label and predicted label of CGM sequences *i*, respectively. We fine-tuned CGMformer towards five specific screening tasks with data from Nationwide Multi-center CGM data and Zhao's CGM data, respectively (for details see Notes S1, 10).

### Subtyping for non-diabetic individuals

We first conduct clustering for the single-day CGM records from samples with NGT or IGR in the Nationwide Multicenter CGM data, annotated as $\mathcal{S}$, to obtain a reference subtyping. The cosine similarity matrix is first calculated, and hierarchal clustering was conducted for the sample based on the similarity matrix. Clusters are named according to the percentage of NGTs and T2Ds in the group. Three clusters Normal, Pre_I, and Pre_II are first classified, and named with increasing ratio of IGRs. In order to obtain better resolution into the subtypes, a more detailed clustering is conducted for the two pre-diabetes clusters. In the end, six clusters, including Normal, Pre_Ia, Pre_Ib, Pre_IIa, Pre_IIb, and Pre_IIc, are identified from the samples.

For a sample *s* to be classified, the vector ${v}_s$ is first calculated from the mean vector of each-day CGM record's embedding from *s*. The sample *s* is classified as the cluster with the highest average cosine similarity to ${v}_s$, that is


\begin{eqnarray*}
T\left( s \right) = \arg \mathop {\max }\limits_{t \in \mathcal{T}} \frac{{\left| {{{\mathrm{\Sigma }}}_{u \in S\left( t \right)}r\left( {{v}_s,{v}_u} \right)} \right|}}{{\left| {S\left( t \right)} \right|}},
\end{eqnarray*}

where $\mathcal{T} = \{ \textit{Normal},Pre\_Ia,Pre\_Ib,Pre\_IIa, Pre\_IIb,Pre\_IIc \}$, $S( t )$ is the set of samples in $\mathcal{S}$ with subtype *t*, ${v}_u$ is the embedding of sample *u*.

### Postprandial glucose prediction

CGMformer_Diet takes the individual's embedding vector from CGMformer, before-meal 1-hour glucose value, and dietary intake as input and output this individual's postprandial 2-hour glucose values (Fig 6a). Formally, three inputs are individual embedding vector encoded from CGMformer ${v}_S \in {\mathbb{R}}^d$; before-meal 1-hour glucose ${G}^B \in {\mathbb{R}}^t$, where *t* is the number of CGM measurements in 1 hour, and for example $t = 4$ for FGM used in Zhao *et al.* [43] which measures glucose every 15 minutes; and the information of the dietary intake, $D = ( {H,C,P,F,B} ) \in {\mathbb{R}}^5$, containing the calories (*H* in unit of *kcal*), carbohydrates (*C* in unit of *g*), proteins (*P* in unit of *g*), fats (*F* in unit of *g*), and dietary fiber (*B* in unit of *g*). CGMformer_Diet predicts postprandial 2-hour glucose ${G}^P \in {R}^{2t}$ as output. The dietary information is encoded as a pulsed perturbation, $\hat{D} \in {\mathbb{R}}^{5 \times t}$, with ${\hat{D}}_{ \cdot .t} = D$ indicating a dietary intake at time *t*, and ${\hat{D}}_{ \cdot ,j} = 0,$ for $j \ne t$, indicating no dietary intake at other time points (Fig S10a). $\hat{D}$ is further concatenated with before-meal glucose ${G}^B$ into $T \in {\mathbb{R}}^{6 \times t}$, with ${T}_{1, \cdot } = {G}^B$ and ${T}_{i, \cdot } = \hat{D}$ for $i \ge 2$.

In CGMformer_Diet, ${v}_s$ is first encoded into a vector $\widehat {{v}_s} \in {\mathbb{R}}^l$ in *l*-dimension latent space for dietary perturbation via a linear encoder ${f}_{enc}$. $\widehat {{v}_s}$ is then adjusted with *T* through LSTM. Specifically, denoting ${v}_1 = \widehat {{v}_s}$, we have


\begin{eqnarray*}
{v}_{k + 1},\ {o}_{k + 1} = \textit{LSTM}\left( {{v}_k,{T}_{ \cdot ,k}} \right)
\end{eqnarray*}

conducted iteratively for $1 \le k \le t$ and results in ${v}_{t + 1}$ for the instant state of sample post dietary perturbation. The outputs, ${o}_{k + 1}$, are decoded to predict the glucose value at $k + 1$ through a linear decoder ${g}_{k + 1} = {f}_{dec}( {{o}_{k + 1}} ) \in \mathbb{R}$.

We then iteratively predict postprandial glucose. Specifically, for $k > t$, we have


\begin{eqnarray*}
{v}_{k + 1},{o}_{k + 1} = \textit{LSTM}\left( {{v}_k,\widehat {{T}_{ \cdot ,k}}} \right),
\end{eqnarray*}

where $\widehat {{T}_{1,k}} = {f}_{dec}( {{o}_k} ),$ indicating the estimated glucose from a previous time, and $\widehat {{T}_{i,k}} = 0$ for $i \ge 1$. The CGMformer_Diet model is optimized by minimizing the following loss function:


\begin{eqnarray*}
{\mathcal{L}}_{{CGMformer}\_Diet} = MSE\left( {{G}_{{pred}},\hat{G}} \right),
\end{eqnarray*}

where ${G}_{pred} = {( {{g}_k} )}_{1 < k \le 3t}$, and $\hat{G} \in {\mathbb{R}}^{3t - 1}$ represents the concatenation of before-meal glucose values $G_{2:t}^B$ and observed postprandial glucose values $\widehat {{G}^P}$. The predicted postprandial glucose ${G}^P$ can be obtained as ${G}^P = {( {{g}_k} )}_{t < k \le 3t}$.

## Supplementary Material

nwaf039_Supplemental_File

## Data Availability

The clinical data and CGM data in the Nationwide Multicenter CGM study and National Real-World CGM study used in this study is available upon request from the corresponding authors. Data in this paper is part of the Human Phenotype Project (HPP) and is accessible to researchers from universities and other research institutions at: https://humanphenotypeproject.org/data-access.

## CODE AVAILABILITY

All code for pretraining and fine-tuning the CGMformer model and downstream tasks are available at: https://github.com/YurunLu/CGMformer.
