# Supplementary material for: A pretrained transformer model for decoding individual glucose dynamics from continuous glucose monitoring data
Source: Natl Sci Rev. 2025 Feb 8;12(5):nwaf039. doi: 10.1093/nsr/nwaf039 (PMC11970253; doi:10.1093/nsr/nwaf039)
Supplement: nwaf039_Supplemental_File [file nwaf039_supplemental_file.docx]

***Supplementary Information for***

**A pretrained transformer model for decoding individual glucose dynamics from continuous glucose monitoring data**

**Contents**

[**Supplementary notes** 2](#_Toc189836612)

[**1.** **Details for Datasets** 2](#_Toc189836613)

[**2.** **Protocol for Nationwide Multicenter CGM data collection** 4](#_Toc189836614)

[**3.** **CGM derived metrics** 5](#_Toc189836615)

[**4.** **TF-IDF adaptive masking** 6](#_Toc189836616)

[**5.** **Baseline methods for masking glucose prediction** 7](#_Toc189836617)

[**6.** **Analyzing long-term association in CGM with auto-regression** 8](#_Toc189836618)

[**7.** **Model training detail** 9](#_Toc189836619)

[**8.** **Model performance evaluation** 9](#_Toc189836620)

[**9.** **Model performance optimization** 10](#_Toc189836621)

[**10.** **Details for finetuning** 12](#_Toc189836622)

[**11.** **Time phase partition** 12](#_Toc189836623)

[**12.** **Identification of HV-NGT as individual with high variant and rapid increasing glucose value** 12](#_Toc189836624)

[**13.** **Contextual attention weights analysis** 12](#_Toc189836625)

[**14.** **Baseline methods for disease screening** 13](#_Toc189836626)

[**15.** **Estimate impairment of glucose regulatory with CGMformer_C** 13](#_Toc189836627)

[**16.** **Dynamic Network Biomarker (DNB) for diabetes risk prediction.** 14](#_Toc189836628)

[**17.** **Glucotype for subtyping** 18](#_Toc189836629)

[**18.** **Prediction of CGMformer_type from CGM derived metrics** 18](#_Toc189836630)

[**19.** **Calculation of Polygenetic Risk Score for genetic risk of diabetes** 18](#_Toc189836631)

[**20.** **Cluster associated SNPs analysis** 19](#_Toc189836632)

[**21.** **In silico dietary perturbation** 19](#_Toc189836633)

[**References** 19](#_Toc189836634)

[**Supplementary tables** 22](#_Toc189836635)

[**Supplementary figures** 30](#_Toc189836636)

**Supplementary notes**

1. **Details for Datasets**

CGMformer is pretrained on two original CGM datasets and finetuned towards various downstream tasks with different internal or external datasets (**Table S4-5**). Specifically, we pretrain CGMformer on two datasets, the **Nationwide multi-center CGM data**[1–3] which includes 964 samples with comprehensive laboratory tests, and the **National Real-World CGM data**[4] which includes 81,800 samples with up to million days of CGM records. CGMformer is further finetuned or validated on several external dataset, including **Zhao’s CGM data**[5], **Colas’s CGM data**[6], **CGMap**[7], **CITY**[8], and **SCENIC**[9]. The details for the datasets are described as follows.

**Nationwide multi-center CGM data:** The nationwide multi-center CGM study enrolled adults from 11 hospitals of 7 provinces and cities in China between 2007 and 2009. This study was independently approved by the ethics committee of each hospital in accordance with the Declaration of Helsinki. Details of the inclusion and exclusion criteria to the study have been described previously. All participants received a complete anthropometric data collection and biochemical measurements, including height, weight, blood pressure, blood glucose and lipid, serum insulin, liver function, and HbA1c. Plasma glucose levels were determined using the glucose oxidase method. HbA1c level was measured by high-performance liquid chromatography on the Variant II HbA1c analyzer (*Bio-Rad Laboratories, Hercules, CA, USA*). Participants recruited to the study were connected to a CGM system sensor (*CGMS GOLD; Medtronic Inc., Northridge, CA, USA*) for 3 consecutive days by the same specialized nurse in each hospital. Participants were instructed and required to enter at least four capillary blood glucose readings (*SureStep; LifeScan, Milpitas, CA, USA*) per day to calibrate the CGM system, with a mean absolute relative deviation (MARD) of 10.17% (**Fig. S1c**). Meanwhile, all participants were required to avoid rigorous physical activities and receive dietary instructions during the CGM. Three daily meals were taken at relatively fixed time. In this study, a total of 964 subjects with complete CGM data were included in our final analysis. According to the World Health Organization 1999 criteria[10,11], normal glucose tolerance was defined as FPG < 6.1 mmol/L and 2hPG < 7.8 mmol/L; prediabetes was defined as 6.1 mmol/L≤ FPG < 7.0 mmol/L, and/or 7.8 mmol/L≤ 2hPG < 11.1 mmol/L; diabetes mellitus was defined as FPG≥7.0 mmol/L, 2hPG≥11.1 mmol/L, and/or HbA1c≥6.5%. In this study, 450 had normal glucose tolerance (NGT), 169 had prediabetes (IGR), and 345 subjects had diabetes (T2D).

**National Real-World CGM data:** We collect National Real-World CGM data, including 1,310,548 days CGM records (*CGM, Shenzhen Sibionics Technology Co. Ltd.*) from 58,847 users from January to December 2022, which reported high accuracy with MARD 8.83%. Data on demographic information, anthropometric indices, and diagnosis of diabetes were collected through standard questionnaires.

**Zhao’s CGM data**: A registry study on Diabetes Data Registry and Individualized Lifestyle Intervention (DiaDRIL) recruited 12 T1DM and 100 T2DM patients in Shanghai East Hospital and Shanghai Fourth People’s Hospital affiliated to Tongji University between 2019 and 2021. Each patient underwent standard questionnaires, physical examination, and information collection from medical records, including diagnosis and treatment of diabetes, duration of diabetes, laboratory measurements, comorbidities and pharmacologic treatments. Meanwhile, all patients wore flash glucose monitoring devices (*FreeStyle Libre H, Abbott Diabetes Care, Witney, UK*) to measure interstitial glucose levels continuously for up to 14 days, and they recorded dietary intakes including the exact time at consumption and weighed food record.

**Colas’s CGM data**: Colas’s CGM data was published in a prospective study conducted in Madrid between 2012 and 2015, targeting at patients considered at increased risk of developing diabetes. The patients wore CGMS devices (*iPro; Medtronics MiniMed, Northridge, CA, USA*) for up to 3 days at inclusion, and then they were followed every 6 months until the diagnosis of T2DM or end of study. A total of 208 patients with a previous diagnosis of essential hypertension from this prospective study were enrolled for external validation.

**CGMap**: CGMap contains CGM data collected from over 7,000 non-diabetic individuals, aged 40–70 years, between 2019 and 2022 to provide reference values of key CGM-derived metrics that can serve as a tool for future CGM research. Data summary in this paper is part of the Human Phenotype Project (HHP) and is accessible to researchers from universities and other research institutions at https://humanphenotypeproject.org/. The raw data is limited in accessing and can only be viewed online through AWS platform.

**CITY**: CITY contains CGM data collected from 74 individuals aged 14 to 24 years with type 1 diabetes. The study is designed to determine the effect of CGM on glycemic control in adolescents and young adults with type 1 diabetes. The raw data was collected from https://public.jaeb.org/dataset/565 .

**SENCE**: SENCE contains CGM data collected from 14 individuals aged 2 to 8 years with type 1 diabetes. The study is designed to evaluate the effects of continuous glucose monitoring (CGM) combined with family behavioral intervention (CGM+FBI) and CGM alone (Standard-CGM) on glycemic outcomes and parental quality of life. The raw data was collected from https://public.jaeb.org/dataset/554 .

1. **Protocol for Nationwide Multicenter CGM data collection**

As reported in our previous studies[1–3], the detailed protocols for CGM in the nationwide multi-center CGM study were as follows.

1. For CGM calibration: Participants were instructed and at least 4 capillary blood glucose readings per day were measured by a SureStep blood glucose meter (*LifeScan, Milpitas, CA, USA*). The criteria for optimal CGM accuracy were adhered to: a correlation between the sensor and meter readings of at least 0.79 and a mean absolute difference of ≤28% (when the daily range [min–max] of meter values was ≥100 mg/dl) and a mean absolute difference of ≤8% (when the daily range [min–max] of meter values was <100 mg/dl). The mean absolute relative deviation (MARD) in this study was 9.20% calculated by sensor glucose and capillary blood glucose.
2. For CGM installation: All the participants volunteered for the retrospective CGM evaluation (*CGMS GOLD; Medtronic Inc., Northridge, CA, USA*) and the systems were inserted by the same specialized nurse in each hospital. First CGM calibration by capillary blood glucose was performed in hospital after 1 h of initialization. If no abnormal CGM-related situation occurred, the subjects then wore the CGM at home for 3 consecutive days (insertion at day 0, removed at day 3).
3. For dietary instruction: All subjects received dietary instructions according to uniform criteria as the CGMS was implemented. The total calorie intake from the three daily meals was 30 kcal/kg/day during CGM, with 50% carbohydrates, 15% proteins, and 35% fats. The amount of drinking water was not restricted. The calorie distribution between breakfast, lunch, and dinner was 20, 40, and 40%, respectively. There was a disciplinary time of 6:30–7:30 A.M. for breakfast, 11:30 A.M. to 12:30 P.M. for lunch, and 6:00–7:00 P.M. for dinner. Each meal had to be consumed within 30 min. The meal time points meals were in accordance with the meal markers of the CGM system input by the participants. If the meal markers were missing, time points were in accordance with the data recorded in each participant’s log book, which guaranteed that there were no missing values. Subjects were required to follow the dietary instruction during the monitoring period.
4. For physical activity: Rigorous physical activities were avoided during the monitoring period.
5. For medication use: Participants with the use of medications that may affect glucose metabolism, such as glucocorticoids, thyroid hormones, and thiazide diuretics, 1 month before the study were excluded. Additionally, none of the participants received any diabetes pharmacotherapy (including metformin) before or during the study.

Taking together, the accuracy of the CGM sensors used in the nationwide multi-center CGM study was comparable to that of the modern sensors. Although there were low accuracy issues of the sensors used in that era (2007 – 2009), the highly controlled conditions ensured the 9.20% MARD value in this study. Of note, the detailed protocols of CGM were carried out in each hospital, which can control and reduce the potential interference of inaccurate methods used, technical issue with the sensor, low sensor accuracy due to bleeding or inflammation, antidiabetic therapy, etc. Thus, the CGM sensor values and glucose dynamics were representative for the “normal” situation, metabolic state, glycemic patterns and fluctuations of participants.

1. **CGM derived metrics**

CGM-derived metrics for glucose control and glucose variability were calculated using the *iglu* R package[12]. Iglu provides an accessible tool to obtain comprehensive CGM evaluation metrics, as opposed to other packages which only provide reading and organizing capabilities or only partial summary measures. The detailed descriptions of CGM-derived measures see **Table S6**. Pairwise Pearson correlations were calculated to characterize the relationships between CGM-derived metrics. The CGM-derived metrics were then clustered using hierarchical clustering based on their correlation (**Figure S2**). As a result, the CGM-derived metrics can be divided into three groups indicting fasting glucose homeostasis, including Mean, eA1C; postprandial glucose adaptation, including SD, MAGE; and others in range and composite metrics, including TIR, J-Index.

1. **TF-IDF adaptive masking**

We utilize a batch-wise adaptive masking strategy in pretraining. Specifically, For each input batch $B$ ($\left| B \right|$= 48*2 in our practice),

$$\mathcal{C =}\left\{ \left[ s_{1},s_{2},\ldots,s_{L} \right]_{s_{k\neq<pad>}}, for s\in B \right\}$$

where $s_{k}$ is $k^{th}$ token in sample $s$, $\mathcal{C}$ represents the set of sequences after removing the $<pad>$ tokens.

The Inverse Document Frequency (IDF) and Term Frequency (TF) for tach token in each sequence from $\mathcal{C}$ is then calculated:

$$DF\left( t \right)=\frac{\left| \left\{ s\in C,t\in s \right\} \right|}{\left| \mathcal{C} \right|}$$

$$IDF\left( t \right)=\log(\frac{\left| \mathcal{C} \right|}{DF\left( t \right)+1})$$

$$TF\left( t,s \right)=\frac{\left| \left\{ i|s_{i}=t \right\} \right|}{\left| s \right|}$$

$$TFIDF\left( t,s \right)=TF\left( t,s \right)\times IDF(t)$$

The masking weights for a token $t$ is than obtained by normalizing the calculated $TFIDF$:

$$W(t)=\frac{\sum_{s\in\mathcal{C}} TFIDF(t,s)}{\max_{r\in\mathcal{T}} (\sum_{s\in\mathcal{C}} TFIDF(r,s))}$$

Where $\mathcal{T}$ is the set of all tokens. The weights are than limited into 0.45-0.6 as a probability for masking token $t$ in batch $B$:

$$P\left( t \right)=\left\{ \begin{aligned} 0.45, &W(t)<0.45 \\ W(t), &0.45\leq W\left( t \right)<0.6 \\ 0.6, &W(t)\geq0.6 \end{aligned} \right.$$

1. **Baseline methods for masking glucose prediction**

We compared CGMformer with various methods for masking glucose prediction. The methods including interpolations such as linear interpolation, KNN (K-nearest neighbors), and STL (Seasonal and Trend decomposition using Loess), and prediction methods such as RC (Reservoir Computing), LSTM (Long short-term memory network), ARIMA (Autoregressive integrated moving average), SVR (Support vector regression), RBF (Radial basis function network) and SVE (Single-variable embedding). The details are as follows:

Linear[13]: Linear interpolation fills in missing data by connecting adjacent data points with a straight line, estimating missing values by assuming a linear progression between known values.

KNN[14]: K-nearest neighbors is a simple classification and regression algorithm that assigns a class or predicts a value based on the majority vote or average of the $k$ closest data points in the feature space. ($k=3$)

STL[15]: Seasonal and Trend decomposition using Loess is a method for decomposing time series data into three components: trend, seasonal, and residual, allowing for better analysis and understanding of each component's behavior. “Seasonal” is used to predict missing values by ARIMA.

RC (Reservoir Computing)[16]: Reservoir computing is a neural network framework that uses a fixed, randomly initialized "reservoir" to process complex dynamic systems. Learning and prediction are achieved by training only a simple output layer, making the training process efficient and straightforward.

LSTM (Long short-term memory network)[17]: Long short-term memory networks are a type of recurrent neural network designed to effectively capture long-term dependencies and temporal patterns in sequential data by using specialized memory cells and gating mechanisms.

ARIMA (Autoregressive integrated moving average)[18]: Autoregressive integrated moving average is a statistical model used for time series forecasting that combines autoregressive terms, integration to handle non-stationarity, and moving average components to capture and predict patterns in data.

SVR (Support vector regression)[19]: Support vector regression is a type of regression model that uses support vector machines to find a function that best fits the data within a specified margin of tolerance, aiming to minimize prediction errors while maximizing generalization.

RBF (Radial basis function network)[20]: Radial basis function networks are a type of artificial neural network that uses radial basis functions as activation functions, typically to perform interpolation and function approximation by mapping inputs to a higher-dimensional space. (para: goal=0.02; spread=1).

SVE (Single-variable embedding)[21]: Single-variable embedding is a forecast model which is based on the weighted average of the nearest neighbors in a single view. The prediction is based on the trend of delay coordinates and only the time series of the target variable is used to make the predictions.

1. **Analyzing long-term association in CGM with auto-regression**

The Autoregressive (AR) model is a time series modeling technique where the value of the series at a particular time point is regressed on its previous values. An AR model of order $p$ can be written as:

$$X_{t}=c+\sum_{i=1}^{p} \phi_{i}X_{t-i}+\varepsilon_{t}$$

where $X_{t}$ is the value of the time series at time $t$, $c$ is a constant term, $p$ is the order of the model, indicating the number of lagged observations included, $\varepsilon_{t}\sim N(0,\sigma^{2})$ is the white noise error term at time $t$, which is assumed to be independently and identically distributed with a mean of zero, ${\theta=\{\phi}_{i}\}$ are the parameters of the model and are optimized to maximize the likelihood function $\mathcal{L}$, which is defined as:

$$\mathcal{L}\left( \theta,\sigma| X \right)=\Pi_{t=p+1}^{n}\frac{1}{\sqrt{2\pi\sigma^{2}}}exp(-\frac{\varepsilon_{t}^{2}}{2\sigma^{2}})$$

An important step in the process in fitting an AR model to CGM data is to select the optimal lag order, which is the number of previous time points that affect the current glucose level. One common method for selecting the optimal lag order is the Akaike Information Criterion (AIC). For each model, the likelihood of the data given the model is computed, and the AIC is calculated using the formula

$$AIC(p)=2k-2ln\mathcal{(L)}$$

where $k$ is the number of parameters and $\mathcal{L}$ is the maximized value of the likelihood function for the model. The optimal order $p$ for observed data $X$ is defined as:

$$p=\arg\min_{p} AIC\left( p \right)$$

In the context of CGM data, we calculate the optimal order of each sample in order to capture the potential long-term association in glucose dynamics. For each sample, the process is conducted 10 times by randomly sampling 10 endpoints from the full time series. The model fitting and parameter optimization is implemented by R package *tseries* and *forecast*.[22,23]

1. **Model training detail**

During pretraining, in each batch of input data, the TF-IDF (term frequency–inverse document frequency) score of each token within the batch is computed and subsequently normalized to determine the actual masking probability, with clipped into 45%~60%. DeepSpeed is used for accelerating during pretraining. Pretraining on the Nationwide Multicenter data is completed in about 3.5 hours on 1 node equipped with 2 Nvidia 3080Ti 12GB GPUs. Pretraining on the National Real-World data is completed in about 5 hours on 1 node equipped with 2 Nvidia A6000 48GB GPUs. Other parameters in pretraining are listed in **Table S7**.

1. **Model performance evaluation**

We evaluate the model performance in both accuracy for masking glucose prediction post pretraining, and accuracy for downstream screening post finetuning.

Regarding the masking glucose prediction accuracy, we calculate MAE for hypoglycemia, hyperglycemia, and euglycemia respectively for evaluate the model comprehensively. Specifically, for masking positions with observed glucose $g_{i}$ and predicted glucose $\hat{g_{i}}$, we calculate following for pretraining stage mode evaluation:

$$MAE_{hypo}=\frac{\left| g_{i}-\hat{g_{i}} \right|_{i\in\mathcal{T}_{mask},g_{i}<70}}{\left| \left\{ i\in\mathcal{T}_{mask}|g_{i}<70 \right\} \right|}$$

$$MAE_{eugly}=\frac{\left| g_{i}-\hat{g_{i}} \right|_{i\in\mathcal{T}_{mask},70\leq g_{i}\leq180}}{\left| \left\{ i\in\mathcal{T}_{mask}|70\leq g_{i}\leq180 \right\} \right|}$$

$$MAE_{hyper}=\frac{\left| g_{i}-\hat{g_{i}} \right|_{i\in\mathcal{T}_{mask},g_{i}>180}}{\left| \left\{ i\in\mathcal{T}_{mask}|g_{i}>180 \right\} \right|}$$

where $\mathcal{T}_{mask}$ is the set of masked glucose tokens. We validate the pretrained model on both the pretraining dataset and several external datasets for external validation.

Regarding the accuracy for downstream screening post finetuning, we finetuned models towards NGT/IGR/T2D screening with Nationwide Multicenter CGM data and conduct 5-fold test for evaluation.

1. **Model performance optimization**

**Loss function.** In CGMformer, the cross-entropy loss is employed to optimize the blood glucose token prediction. We also adopted MAE loss to pretraining the model for comparison, which is defined as follows:

$$\mathcal{L}_{tokens}=\Sigma_{j\in\mathcal{T}_{mask}}|g_{j}-\hat{g_{j}}|/|\mathcal{T}_{mask}|$$

where $\mathcal{T}_{mask}$ denotes the set of masked tokens, $\hat{g_{j}}$ denotes the predicted glucose, $g_{j}$ denotes the real glucose.

**Model architectures.** We compared our encoder-only model with two other architectures: decoder-only and encoder-decoder. For the decoder-only architecture, we utilized the Llama2 model from the *Huggingface Transformers* library to pretrain the data in an autoregressive manner. This model was implemented with 4-layer decoders, 8 attention heads per layer, and 128-dimensional latent space. For the encoder-decoder architecture, we used the BART model from the *Huggingface Transformers* library, which employs bidirectional attention in the encoder and unidirectional attention in the decoder. This model was implemented with 2-layer encoders and 2-layer decoders, 8 attention heads per layer, and 128-dimensional latent space. Training parameters were kept consistent during the training phase.

**Hidden space dimension.** We evaluated the effect of three hidden space dimensions (dim=32, 64, 128) on the reconstruction accuracy of masked tokens during the training phase. For clinical medical significance, we classified the masked tokens into three categories based on the level of blood glucose values, including normal glycemia (70-180 mg/dl), hyperglycemia (>180 mg/dl), and hypoglycemia (<70 mg/dl). The highest reconstruction accuracy is achieved on all three types of tokens when the hidden space dimension is taken as 128 (**Fig. S3a**). We further consider the performance of different hidden space representation dimensions on downstream multiclassification tasks. To evaluate whether larger hidden space dimensions lead to better performance, we consider four hidden space dimensions (dim=32, 64, 128, 256), and find that the hidden space dimension 128 is slightly lower than 64, but higher than the others for the metrics on the multiclassification tasks. Notably, when the hidden space dimension is 256, no better results are achieved (**Fig. S3b**). To balance token- and sample-level performance, we chose a hidden layer dimension of 128, achieving favorable results in both pre-training and downstream applications, particularly in clinical medicine.

**Masking rate and masking strategy.** We explored different masking rates and masking strategies with our practice. For masking rate, we compared experiments with masking rates of 15%, 45%, and 60%, we observed increased representational capacity and label classification accuracy as the rate increased from 15% to 45%. However, further increasing the rate to 60% led to a decline in representational capacity (**Fig. S3c**). We targeted a masking rate within the 45%-60% range. Additionally, considering the clinical significance of CGM data, we implemented an adaptive masking strategy based on each token's TF-IDF value, normalized and truncated within the 45%-60% range, which results in a higher mask probability for hyperglycemia and hypoglycemia tokens. Random sampling within this range served as a control experiment, verifying the effectiveness of our adaptive masking strategy in enhancing label classification performance (**Fig. S3c-d**). We also conducted comparison for token-wise masking with patch-wise masking strategy. Specifically, we first sampling patch center randomly or adaptively, and extend the center to bi-direction to obtain a N-tokens long patch mask.

**Input sequence length.** Accommodating and harmonizing diverse CGM data formats was a key challenge in our study. Different devices monitor blood glucose at varying intervals, resulting in day-long data lengths of 96 or 288 points. Since our nationwide multi-center data originated from 5-minute monitoring devices, we down-sampled the 288-point sequences to 144, 96, and 48 points (Specifically, we average the values of the three time points in the sequence of length 288 to obtain a point in the corresponding sequence of length 96). Each down-sampled sequence was then used to train a pre-trained model with identical parameters. The dataset with a length of 288 (implying more frequent sampling) achieves the best results in label prediction for NGT/IGR/T2D (**Fig. S3e**), while the model remains capacity for label prediction after input down-sampling, illustrating the generality of the pretrained model.

**Pretraining data volume.** To investigate the relationship between pre-training data size and downstream tasks performance, we trained CGMformer models on six datasets of varying sizes, ranging from 250 to 1,917 samples (250, 450, 750, 1,150, 1,650, and 1,917), subsampling randomly from our nationwide multi-center CGM study.

**Data downsampling.** To investigate the robustness of model for data missing or across devices, we down sampled the input data every 6 or 3 (for simulate devices with time interval of 30/15 minutes) measurements. The vacancy positions were imputed with <PAD> token.

1. **Details for finetuning**

**Label classification (NGT/IGR/T2D screening) in Nationwide Multi-center CGM data.** We finetuned the pretrained CGMformer on the nationwide multicenter data with it diagnosed labels (NGT/IGR/T2D). We divided the samples from Nationwide Multicenter CGM data into five groups randomly to perform a 5-fold test.

**T1D/T2D and complication screening in Zhao’s dataset.** We finetuned the pretrained CGMformer on the Zhao’s CGM data[5] with four labels including it diagnosed labels (T1D/T2D), and complications (with/without complications, macrovascular, or microvascular). Specifically, CGM data in Zhao’s dataset is 15-min interval, and we performed linear interpolation for the raw data to obtain a data with 5-min interval. We divided the samples from Zhao’s CGM data into five groups randomly to perform a 5-fold test.

1. **Time phase partition**

**Fasting phase.** For samples with recorded meal intake times, fasting phases are defined as the period starting at least 2 hours after dinner intake on the last day until the subsequent breakfast intake. In cases where meal intake times are available, fasting phases are alternatively defined as the time interval from 0:00 to 6:00.

**Postprandial phase.** postprandial phases are characterized as the duration spanning from meal intake to 2 hours after the meal intake.

1. **Identification of HV-NGT as individual with high variant and rapid increasing glucose value**

We choose the individual with high variant and rapid increasing glucose value by calculating two indexes, Fasting SD $SD_{fast}$ and glycemic rate $G_{r}$, from CGM records for samples. Fasting SD is calculated as standard deviation during fasting phase, that is $SD_{fast}=SD(\left[ g_{t} \right]_{t in fasting phase})$. Glycemic rate is calculated as $G_{r}=(g_{max}-g_{0})/T$, where $g_{max}$ is the maximum glucose after meal intake, $g_{0}$ is the glucose before meal intake, $T$ is the time interval to reach the maximum after meal intake. HV-NGT are identified as individuals with both top 25% $SD_{fast}$ and $G_{r}$ in NGTs.

1. **Contextual attention weights analysis**

Each of CGMformer’s 4-layer attention blocks has 8 self-attention heads that are meant to learn in an unsupervised manner to pay attention to distinct classes of blood glucose value to jointly improve predictions. Contextual CGMformer attention weights are extracted for each attention head within each self-attention layer for each blood glucose value within the given CGM sequences evaluated by forward pass through the CGMformer model.

1. **Baseline methods for disease screening**

**ML-base methods for disease screening:** The LSTM model was implemented using PyTorch with 3 layers, 64 hidden dimensions, and a learning rate of 0.005. The MLP model was implemented using scikit-learn with a three-layer architecture and node configurations of (128, 64, 1). We employed grid search and cross-validation to identify the optimal set of parameters for both models. For the LSTM model, we explored various configurations, including different numbers of layers (1-4), hidden dimensions (32, 64, 128), and learning rates (0.001, 0.005, 0.01). Similarly, for the MLP model, we tested different numbers of nodes in each layer, learning rates, and regularization parameters.

**CGM derived metrics-based screening:** Samples are diagnosed as T2D if meeting the following thresholds according to Zhou et al.[1]:

- Mean: Mean$\geq$6.6 mmol/L
- SD: SD$\geq$1.4 mmol/L
- MAGE: MAGE$\geq$3.9 mmol/L
- TAR: Time above range (>140 mg/dL)$\geq$17%
- TIR: Time in range (70~140 mg/dL)$\leq$70%

**Combing CGM-derived metrics for screening:** We employ six machine-learning models, including Ridge Regression, Linear Regression, Logistic Regression, XGBoost, MLP, and Randon Forest, for disease screening by integrating CGM-derived metrics calculated by *iglu*. The models are implemented using *scikit-learn* with default parameters.

1. **Estimate impairment of glucose regulatory with CGMformer_C**

CGMformer_C takes the CGMformer encoded vector $v_{s}$ as input and outputs a value C between 0 and 1. It is designed with an encoder which encode the input vector into a value C between 0 and 1, and a decoder which links the value C with labels including clinical information and diagnosis. The encoder is composed of three linear layers followed with a nonlinear sigmoid activation layer, and encoded the input vector $v_{s}\in\mathbb{R}^{d}$ in to $C\in(0,1)$:

$$C=Encoder\left( v_{s} \right)\in(0,1)$$

The decoder conducts multitask prediction, including prediction for the labels. For the prediction of label, the standard index $I$ is defined as 0 for NGT, 0.5 for IGR, and 1 for T2D. We further decode $C$ into multi-task prediction with a linear layer followed with an activation function to predicting clinical information, including age, BMI, HbA1c, FPG, PG120, Ins, Ins120:

$$M=Decoder\left( C \right)\in\mathbb{R}^{N}$$

where $N$ is the number of predicted targets.

The parameter in CGMformer_C model is optimized to minimize the loss function:

$$\mathcal{L}_{CGMformer\_C}=p\cdot MSE\left( C,I \right)+\left( 1-p \right)\cdot MSE(M,\hat{M})$$

where $\hat{M}$ is individual clinical information, $p$ is parameter to balance the loss of diagnosis prediction and clinical information regression. The encoded value, $C$, is defined as the CGMformer derived course of diabetes, indicating the impairment of individual glucose regulatory.

1. **Dynamic Network Biomarker (DNB) for diabetes risk prediction.**

We introduce the theoretical background of the DNB theory. Firstly, we consider the following discrete-time dynamical system that represents the dynamic evolution of a district network:

$Z\left( k+1 \right)=f\left( Z\left( k \right);P \right),$ (1)

where $Z\left( k \right)=\left( z_{1}(k), \ldots, z_{n}(k) \right)$ is an $n$-dimensional state vector or variables at time instant $k$ that represents districts or cities in the system, while $P=\left( p_{1}, \ldots, p_{s} \right)$ is a parameter vector or driving factors that represent slowly changing/evolution factors. Mappings $f:\mathbb{R}^{n}\times\mathbb{R}^{s}\to\mathbb{R}^{n}$ are generally nonlinear functions. For such dynamic evolution function Eq. (1) with the parameter *P*, there is a bifurcation or critical state if some transversal conditions hold.

1. $\bar{Z}$ is a fixed point of system such that $\bar{Z}=f(\bar{Z};P)$.
2. There is a value $P_{c}$ such that one or a pair of the eigenvalues of the Jacobian matrix $\left. \frac{\partial f\left( Z;P_{c} \right)}{\partial Z} \right|_{Z=\bar{Z}}$ is equal to 1 in the modulus. And this Jacobian matrix is diagonalizable.
3. When $P\neq P_{c}$, the eigenvalues of the linearized function of $f$ are not always equal to 1 in the modulus.

The above three conditions with other transverse conditions imply that the system undergoes a phase change at $\bar{Z}$ or a bifurcation when $P$ reaches the threshold$P_{c}$. For system Eq. (1) near $\bar{Z}$and before $P$ reaches $P_{c}$, we assume that the system is at a hyperbolic stable fixed point $\bar{Z}$ and thus all of the eigenvalues are within (0, 1) in modulus. The parameter value $P_{c}$ at which the state shift of the system occurs, is known as a bifurcation parameter value or a critical transition value.

Assuming that the state approaches a saddle-node bifurcation point for the case of simplicity, the generic properties in dynamics of Eq. (1) was theoretically derived by introducing new variables $Y\left( k \right)=\left( y_{1}(k), \ldots, y_{n}(k) \right)$ and a transformation matrix $S=\{s_{ij}\}$, such that

$Y\left( k \right)=S^{-1}(Z\left( k \right)-\bar{Z})$. (2)

With the variable transformation Eq. (2), a linearized form of the original system Eq. (1) is presented as

$Y\left( k+1 \right)=\Lambda\left( P \right)Y\left( k \right)+\zeta\left( k \right),$ (3)

where $\Lambda(P)$ is the diagonalized matrix of $\left. \frac{\partial f\left( Z;P_{c} \right)}{\partial Z} \right|_{Z=\bar{Z}}$, and $\zeta\left( k \right)=\left( \zeta_{1}(k), \ldots, \zeta_{n}(k) \right)$ are small Gaussian noises with zero means and the covariance $\mathrm{COV}(\zeta_{i},\zeta_{j})=\kappa_{ij}$. The norm ${|\lambda}_{i}|$ in the diagonalized matrix $\Lambda\left( P \right)=diag(\lambda_{1}(P),\ldots,\lambda_{n}(P))$ is between 0 and 1, implying that the system is at a stable state initially. Thus, for the standard deviation and covariance of abstract variable $y_{i}$, there are the following expressions [1].

$\mathrm{SD}\left( y_{i} \right)=\sqrt{\frac{\kappa_{ii}}{1-\lambda_{i}^{2}}} ,$ (4)

$\mathrm{COV}\left( y_{i},y_{j} \right)=\frac{\kappa_{ij}}{1-\lambda_{i}\lambda_{j}} .$ (5)

Denote the dominant eigenvalue as the largest eigenvalue or eigenvalues (the case of multiple roots) in modulus, which characterize the system’s rate of change around a fixed point. Then, the before-transition/normal stage corresponds to a period when the dominant eigenvalue is far smaller than 1 in modulus (as parameter $P$ is far away from $P_{c}$). The critical stage corresponds to the period when the dominant eigenvalue approaches to 1 in modulus (as parameter $P\to P_{c}$). Clearly, when the dominant eigenvalue ${|\lambda}_{1}|\to+\infty$, there is $\mathrm{SD}\left( y_{1} \right)\to+\infty$.

Back to the original system of $z$, when the largest eigenvalue (or eigenvalue pairs) approaches 1 in modulus, there are three generic codimension-one bifurcations of the system (i.e., the saddle-node, period-doubling, and Neimark-Sacker bifurcations). Specifically, when the dominant eigenvalue is real, the critical point is the saddle-node bifurcation (transcortical and pitchfork) if the dominant eigenvalue approaches 1, while the critical point is the period-doubling (or flip) bifurcation if the dominant eigenvalue approaches -1. When the dominant eigenvalues are a pair of complex conjugate eigenvalues (including several pairs with the same modulus), the critical point is the Neimark-Sacker bifurcation. According to our previous work [1-3], we have the following results.

1. When the dominant eigenvalue $\lambda_{1}$ is real, there is only one dominant group related to variable $y_{1}$ in Eq. (3). Among this dominant group, each variable $z_{i}$ has nonzero coefficient $s_{i1}\neq0$ in the transformation matrix $S=\{s_{ij}\}$ of Eq. (2), and has standard deviation (SD)

$\mathrm{SD}\left( z_{i} \right)=\sqrt{s_{i1}^{2}\frac{\kappa_{11}}{1-\lambda_{1}^{2}}+\sum_{l=2}^{n} s_{il}^{2}\frac{\kappa_{ll}}{1-\lambda_{l}^{2}}+\sum_{l,m=1,l\neq m}^{n} s_{il}s_{im}\frac{\kappa_{lm}}{1-\lambda_{l}\lambda_{m}}},$ (6)

and Pearson correlation coefficient (PCC) with other variables $z_{j}$

$\mathrm{PCC}\left( z_{i},z_{j} \right)=\frac{s_{i1}s_{j1}\frac{\kappa_{11}}{1-\lambda_{1}^{2}}+\sum_{l=2}^{n} s_{il}s_{jl}\frac{\kappa_{ll}}{1-\lambda_{l}^{2}}+\sum_{l,m=1,l\neq m}^{n} s_{il}s_{jm}\frac{\kappa_{lm}}{1-\lambda_{l}\lambda_{m}}}{\sqrt{\sum_{l=1}^{n} \frac{s_{il}^{2}\kappa_{ll}}{1-\lambda_{l}^{2}}+\sum_{l,m=1,l\neq m}^{n} \frac{s_{il}s_{im}\kappa_{lm}}{1-\lambda_{l}\lambda_{m}}}\sqrt{\sum_{l=1}^{n} \frac{s_{jl}^{2}\kappa_{ll}}{1-\lambda_{l}^{2}}+\sum_{l,m=1,l\neq m}^{n} \frac{s_{jl}s_{jm}\kappa_{lm}}{1-\lambda_{l}\lambda_{m}}}},$ (7)

For this case, when parameter$P\to P_{c}$, $\mathrm{SD}\left( z_{i} \right)$ increases sharply, and $|\mathrm{PCC}\left( z_{i},z_{j} \right)|$ increases to 1 (if $z_{j}$ is also in this dominant group).

2. When the dominant eigenvalues $\lambda_{1}=a+ib$ and $\lambda_{2}=a-ib$ are a pair of complex conjugate eigenvalues, then there are two dominant groups respectively related to $y_{1}$ and $y_{2}$ in Eq. (3). In each of the dominant group, a variable $z_{i}$ has standard deviation

$\mathrm{SD}\left( z_{i} \right)=\sqrt{\frac{2b^{2}(s_{i1}^{2}+s_{i2}^{2})(\kappa_{11}+\kappa_{22})}{(1-a^{2}-b^{2})(\left( a-1 \right)^{2}+b^{2})(\left( a+1 \right)^{2}+b^{2})}+K_{i}},$ (8)

where $K_{i}$ is a bounded value, and Pearson correlation coefficient with other variable $z_{j}$

$\mathrm{PCC}\left( z_{i},z_{j} \right)=\frac{\frac{2b^{2}(s_{i1}s_{j1}+s_{i2}s_{j2})(\kappa_{11}+\kappa_{22})}{(1-a^{2}-b^{2})(\left( a-1 \right)^{2}+b^{2})(\left( a+1 \right)^{2}+b^{2})}+C_{ij}}{\sqrt{\frac{2b^{2}(s_{i1}^{2}+s_{i2}^{2})(\kappa_{11}+\kappa_{22})}{(1-a^{2}-b^{2})(\left( a-1 \right)^{2}+b^{2})(\left( a+1 \right)^{2}+b^{2})}+K_{i}}\sqrt{\frac{2b^{2}(s_{j1}^{2}+s_{j2}^{2})(\kappa_{11}+\kappa_{22})}{(1-a^{2}-b^{2})(\left( a-1 \right)^{2}+b^{2})(\left( a+1 \right)^{2}+b^{2})}+K_{j}}},$ (9)

where $C_{ij}$, $K_{i}$ and $K_{j}$ are bounded values. In each dominant group, when parameter$P\to P_{c}$, both $\mathrm{SD}\left( z_{i} \right)$ and $|\mathrm{PCC}\left( z_{i},z_{j} \right)|$ increases sharply (if $z_{j}$ is also in the same dominant group).

For the original system Eq. (1), when the dynamical system approaches a critical point (bifurcation point), at least a dominant group appears and can be defined as the dynamic network biomarker (DNB), which meets the following two necessary conditions in terms of the observed data with noise.

(1) The coefficient of variation for any element in the DNB group rapidly increases.

(2) The absolute Pearson's correlation coefficient between the DNB elements rapidly increases.

The above two conditions are generic properties of the state transition at a tipping point, which can be approximately stated as: the occurrence of a strongly fluctuating and highly correlated group of elements implies an upcoming transition into the after-transition stage.

According to the DNB theory, for a discrete-time dynamical system, the appearance of a strongly fluctuating and highly correlated group of elements implies a tipping point or an upcoming transition into the after-transition stage[24–26]. In other words, critically collective fluctuation of a group of elements means the imminent critical transition. The DNB conditions were computed to assess if a subject is going to experience a critical transition into T2D. Temporal segmentation of CGM data: for each subject and each day, the CGM time-series data were segmented according to the following rules:

- Segmentation was performed based on the three main meals: breakfast, lunch, and dinner.
- Meal times were determined using local maxima in the CGM data. Breakfast: within the time range 6:00 to 9:00, identified by the local maximum T1, corresponding to the period [T1-3h, T1+1h]. Lunch: within the time range 11:00 to 14:00, identified by the local maximum T2, corresponding to the period [T2-3h, T2+1h]. Dinner: within the time range 17:00 to 21:00, identified by the local maximum T3, corresponding to the period [T3-3h, T3+1h].
- Additionally, two segments were added for the early morning (2:00 to 6:00) and fasting (21:00 to 1:00 of the next day) periods on the first day. Consequently, each subject's CGM time-series was divided into eight segments, each containing continuous CGM data measured over a 4-hour interval, representing a 48-dimensional vector.

The standard deviation SD for CGM data within each of the eight time-segments were computed for every subject, and the average variance across all segments was obtained

1. **Glucotype for subtyping**

Glucotype[27] first classify different patterns of glycemic responses based on their variability with spectral clustering, obtain fraction of time with low/moderate/severe variability, and classify individuals into low/moderate/severe variability groups. We utilize Glucotype for sample subtyping with the released code at <https://github.com/abreschi/shinySpecClust>. We compared Glucotype with our CGMformer based subtyping.

1. **Prediction of CGMformer_type from CGM derived metrics**

Access to CGM data in CGMap is limited to an online platform where we cannot directly install CGMformer. To annotate samples in CGMap with CGMformer_type, we trained a random forest classifier to predict CGMformer types from the 48 CGM-derived metrics. The classifier was trained on the Nationwide Multicenter CGM data, utilizing paired metrics and subtype labels. We then applied this classifier to CGMap to obtain annotations for the samples in CGMap.

1. **Calculation of Polygenetic Risk Score for genetic risk of diabetes**

We collected 1,289 T2D association signals from Suzuki et al.[28] For a sample $s$, its Polygenetic Risk Score (PRS) is calculated by:

$$PRS\left( s \right)=\Sigma_{v\in V}\beta_{v}g_{v}(s)$$

where $V$ is the SNPs set, $\beta_{v}=\log OR_{v}$ denotes the effect size of SNP $v$, and $g_{v}\left( s \right)\in\{0,1,2\}$ denotes the genotype of SNP $v$ in sample $s$. The SNPs are classified into eight clusters according to Suzuki et al.[28] We further calculated the cluster specific PRS for samples:

$$PRS_{c}\left( s \right)=\Sigma_{v\in V_{c}}\beta_{v}g_{v}(s)$$

where $V_{c}$ denotes the set of SNPs in the cluster $c$.

1. **Cluster associated SNPs analysis**

We conducted association study for the 1,289 SNPs from Suzuki et al.[28] with CGMformer_type. Specifically, each subtype was treated as a binary phenotype (presence or absence), and chi-square test was performed for each SNP.

1. **In silico dietary perturbation**

For each dietary recorded in Zhao et al. [5], we established a standard balanced dietary with fixed calories and an energy supply ratio of carbohydrate: protein: fat = 5:2:3. To assess the glucose response to different dietary intake plan, we designed three additional simulated meal plans with adjusted ratios—low carbohydrate-high protein (carbohydrate: protein: fat = 4:3:3), low carbohydrate-high fat (carbohydrate: protein: fat = 4:2:4), and high protein (carbohydrate: protein: fat = 5:3:2). The calories for carbohydrate, protein and fat are estimated as: carbohydrate: $4 kcal/g$, protein: $4 kcal/g$, fat: $9 kcal/g$.

Specifically, for a raw meal recorded with $H (kcal)$ calories, $C (g)$ carbohydrate, $P (g)$ protein, $F (g)$ fats, and $B (g)$ dietary fiber, $D_{raw}=(H,C,P,F,B)$, simulated dietarys are defined as:

Standard: $D_{standard}=\left( H,0.5*H/4,0.2*H/4,0.3*H/9,B \right)$

Low carbohydrate and high protein: $D_{Car_{L}Pro_{H}}=\left( H,0.4*H/4,0.3*H/4,0.3*H/9,B \right)$

Low carbohydrate and high fats: $D_{Car_{L}Fat_{H}}=\left( H,0.4*H/4,0.2*H/4,0.4*H/9,B \right)$

Common carbohydrate and high protein: $D_{Pro_{H}}=\left( H,0.5*H/4,0.3*H/4,0.2*H/9,B \right)$

**References**

1. Zhou J, Li H, Ran X *et al.* Reference values for continuous glucose monitoring in Chinese subjects. *Diabetes care* 2009;**32**:1188–93.

2. Zhou J, Mo Y, Li H *et al.* Relationship between HbA1c and continuous glucose monitoring in Chinese population: a multicenter study. *PloS one* 2013;**8**:e83827.

3. Cheng Li XM. Decreasing complexity of glucose time series derived from continuous glucose monitoring is correlated with deteriorating glucose regulation. *Front Med* 2023;**17**:68–74.

4. Yan L, Li Q, Guan Q *et al.* Evaluation of the performance and usability of a novel continuous glucose monitoring system. *Int J Diabetes Dev Ctries* 2023;**43**:551–8.

5. Zhao Q, Zhu J, Shen X *et al.* Chinese diabetes datasets for data-driven machine learning. *Sci Data* 2023;**10**:35.

6. Colás A, Vigil L, Vargas B *et al.* Detrended Fluctuation Analysis in the prediction of type 2 diabetes mellitus in patients at risk: Model optimization and comparison with other metrics. *PloS one* 2019;**14**:e0225817.

7. Keshet A, Shilo S, Godneva A *et al.* CGMap: Characterizing continuous glucose monitor data in thousands of non-diabetic individuals. *Cell Metabolism* 2023;**35**:758-769.e3.

8. Laffel LM, Kanapka LG, Beck RW *et al.* Effect of Continuous Glucose Monitoring on Glycemic Control in Adolescents and Young Adults With Type 1 Diabetes: A Randomized Clinical Trial. *JAMA* 2020;**323**:2388.

9. Strategies to Enhance New CGM Use in Early Childhood (SENCE) Study Group, Laffel L, Harrington K *et al.* A Randomized Clinical Trial Assessing Continuous Glucose Monitoring (CGM) Use With Standardized Education With or Without a Family Behavioral Intervention Compared With Fingerstick Blood Glucose Monitoring in Very Young Children With Type 1 Diabetes. *Diabetes Care* 2021;**44**:464–72.

10. Consultation WHO. *Definition, Diagnosis and Classification of Diabetes Mellitus and Its Complications*. Part, 1999.

11. *Use of Glycated Haemoglobin (HbA1c) in the Diagnosis of Diabetes Mellitus: Abbreviated Report of a WHO Consultation*. Geneva: World Health Organization, 2011.

12. Broll S, Urbanek J, Buchanan D *et al.* Interpreting blood GLUcose data with R package iglu. Pyle L (ed.). *PLoS ONE* 2021;**16**:e0248560.

13. Bartholomew DJ, Box GEP, Jenkins GM. Time Series Analysis Forecasting and Control. *Operational Research Quarterly (1970-1977)* 1971;**22**:199.

14. Murti DMP, Pujianto U, Wibawa AP *et al.* K-Nearest Neighbor (K-NN) based Missing Data Imputation. *2019 5th International Conference on Science in Information Technology (ICSITech)*. Yogyakarta, Indonesia: IEEE, 2019, 83–8.

15. Cleveland RB, Cleveland WS, McRae JE *et al.* STL: A seasonal-trend decomposition. *J off Stat* 1990;**6**:3–73.

16. Verstraeten D, Schrauwen B, D’Haene M *et al.* An experimental unification of reservoir computing methods. *Neural Networks* 2007;**20**:391–403.

17. Hochreiter S, Schmidhuber J. Long short-term memory. *Neural computation* 1997;**9**:1735–80.

18. Box GE, Pierce DA. Distribution of residual autocorrelations in autoregressive-integrated moving average time series models. *Journal of the American statistical Association* 1970;**65**:1509–26.

19. Kecman V, Huang T-M, Vogt M. Iterative single data algorithm for training kernel machines from huge data sets: Theory and performance. *Support vector machines: Theory and Applications* 2005:255–74.

20. Orr MJ, others. Introduction to radial basis function networks. 1996.

21. Sugihara G, May RM. Nonlinear forecasting as a way of distinguishing chaos from measurement error in time series. *Nature* 1990;**344**:734–41.

22. Trapletti A, Hornik K. tseries: Time Series Analysis and Computational Finance. 1999:0.10-57.

23. Hyndman R, Athanasopoulos G, Bergmeir C *et al.* forecast: Forecasting Functions for Time Series and Linear Models. 2009:8.23.0.

24. Chen L, Liu R, Liu Z-P *et al.* Detecting early-warning signals for sudden deterioration of complex diseases by dynamical network biomarkers. *Scientific reports* 2012;**2**:342.

25. Liu R, Chen P, Aihara K *et al.* Identifying early-warning signals of critical transitions with strong noise by dynamical network markers. *Scientific reports* 2015;**5**:17501.

26. Liu R, Zhong J, Hong R *et al.* Predicting local COVID-19 outbreaks and infectious disease epidemics based on landscape network entropy. *Science Bulletin* 2021;**66**:2265–70.

27. Hall H, Perelman D, Breschi A *et al.* Glucotypes reveal new patterns of glucose dysregulation. *PLOS Biology* 2018;**16**:e2005143.

28. Suzuki K, Hatzikotoulas K, Southam L *et al.* Genetic drivers of heterogeneity in type 2 diabetes pathophysiology. *Nature* 2024, DOI: 10.1038/s41586-024-07019-6.

**Supplementary tables**

**Table S1. Statistics for nationwide multi-center CGM study.**

|  | NGT | IGR | T2D |
| --- | --- | --- | --- |
| Age | 43.29 (14.37) | 55.79 (10.95) | 53.04 (11.87) |
| BMI (kg/m^2^) | 22.11 (2.09) | 24.47 (3.08) | 25.82 (3.64) |
| LDL-C (mmol/l) | 2.72 (0.84) | 2.99 (0.87) | 3.09 (0.94) |
| HDL-C (mmol/l) | 1.56 (0.40) | 1.33 (0.43) | 1.23 (0.30) |
| HbA1c (%) | 5.51 (0.46) | 5.81 (0.38) | 7.58 (1.50) |
| FPG (mg/dl) | 86.35 (7.93) | 104.89 (10.87) | 141.12 (42.98) |
| PG120 (mg/dl) | 96.95 (20.69) | 154.12 (28.02) | 263.80 (84.96) |
| INS0 (μU/ml) | 11.30 (4.38) | 13.78 (7.00) | 16.08 (10.31) |
| INS120 (μU/ml) | 46.06 (31.89) | 83.40 (66.90) | 79.66 (65.73) |
| HOMA-β | 173.13 (82.40) | 128.11 (79.23) | 90.08 (63.99) |
| HOMA-IS | 0.47 (0.20) | 0.35 (0.18) | 0.27 (0.25) |

**Table S2. Characteristics of CGMformer_types with participants from the nationwide multicenter cohort.**

|  | **Subtypes of non-diabetes stratified by CGMformer_type** | | | | | | **Diabetes** |
| --- | --- | --- | --- | --- | --- | --- | --- |
|  | **Normal** | **Pre_Ia** | **Pre_Ib** | **Pre_IIa** | **Pre_IIb** | **Pre_IIc** |  |
| **Num** | 58 | 205 | 112 | 147 | 47 | 50 | 345 |
| **Age (Mean (std))** | 37.89 (12.53) | 44.22 (14.25) | 44.68 (15.82) | 50.99 (13.05) | 55.00 (11.78) | 47.74 (14.84) | 53.04 (11.87) |
| **Female (n, %)** | 24 (41.38%) | 102 (49.76%) | 58 (51.79%) | 78 (53.06%) | 20 (42.55%) | 23 (46.00%) | 152 (44.06%) |
| **Glycemic status** | | | | | | | |
| NGT (n, %) | 52 (89.66%) | 172 (83.90%) | 93 (83.04%) | 88 (59.86%) | 12 (25.53%) | 33 (66.00%) | 0 (0.00%) |
| IGT (n, %) | 2 (3.45%) | 15 (7.32%) | 8 (7.14%) | 33 (22.45%) | 17 (36.17%) | 7 (14.00%) | 0 (0.00%) |
| IFG (n, %) | 0 (0.00%) | 5 (2.44%) | 1 (0.89%) | 8 (5.44%) | 4 (8.51%) | 0 (0.00%) | 0 (0.00%) |
| CGI (n, %) | 1 (1.72%) | 7 (3.41%) | 5 (4.46%) | 15 (10.20%) | 14 (29.79%) | 7 (14.00%) | 0 (0.00%) |
| ***Anthropometric and laboratory tests (Mean (std))*** | | | | | | | |
| BMI (kg/m^2^) | 21.59 (1.85) | 22.31 (2.23) | 22.27 (2.48) | 23.30 (2.65) | 25.07 (3.26) | 22.59 (2.44) | 25.82 (3.64) |
| SBP (mmHg) | 112.49 (10.70) | 115.85 (14.42) | 115.63 (13.35) | 120.13 (14.99) | 125.56 (14.94) | 116.40 (15.48) | 128.46 (16.97) |
| TC (mmol/l) | 4.56 (1.00) | 4.60 (0.85) | 4.70 (0.99) | 4.96 (0.95) | 5.06 (1.23) | 4.47 (0.85) | 5.15 (1.08) |
| LDL-C (mmol/l) | 2.55 (0.82) | 2.73 (0.75) | 2.70 (0.91) | 3.00 (0.88) | 2.98 (0.96) | 2.69 (0.92) | 3.09 (0.94) |
| HDL-C (mmol/l) | 1.69 (0.52) | 1.48 (0.39) | 1.57 (0.40) | 1.49 (0.41) | 1.35 (0.40) | 1.40 (0.42) | 1.23 (0.30) |
| ALT (U/l) | 17.62 (9.54) | 20.23 (11.91) | 17.81 (10.34) | 22.23 (12.19) | 25.17 (14.09) | 18.58 (10.30) | 32.26 (19.28) |
| FPG (mg/dl) | 83.81 (7.56) | 90.55 (9.74) | 86.03 (10.40) | 94.81 (12.64) | 103.40 (13.42) | 91.57 (12.10) | 141.12 (42.98) |
| PG30 (mg/dl) | 138.34 (31.15) | 145.61 (28.45) | 141.31 (31.42) | 162.59 (35.25) | 173.05 (34.04) | 144.88 (29.63) | 232.28 (52.14) |
| PG60 (mg/dl) | 126.97 (41.15) | 131.97 (42.99) | 125.14 (45.47) | 160.95 (56.80) | 181.42 (55.44) | 142.14 (45.44) | 277.03 (62.32) |
| PG120 (mg/dl) | 96.79 (23.56) | 106.00 (28.19) | 99.49 (27.25) | 123.98 (36.14) | 142.91 (38.14) | 113.22 (37.68) | 263.80 (84.96) |
| PG180 (mg/dl) | 72.22 (20.36) | 78.89 (20.55) | 73.08 (23.13) | 85.75 (26.91) | 94.04 (31.67) | 81.21 (26.19) | 175.39 (81.69) |
| INS0 (μU/ml) | 9.91 (3.84) | 11.64 (4.51) | 10.86 (4.74) | 13.06 (5.91) | 15.49 (7.35) | 9.90 (3.50) | 16.08 (10.31) |
| INS30 (μU/ml) | 58.02 (26.98) | 66.61 (35.82) | 77.54 (81.81) | 72.16 (43.37) | 66.59 (43.81) | 51.09 (28.85) | 47.93 (35.13) |
| INS60 (μU/ml) | 45.12 (15.82) | 76.91 (48.03) | 70.98 (43.52) | 87.97 (56.60) | 86.15 (48.68) | 61.49 (34.60) | 76.95 (56.33) |
| INS120 (μU/ml) | 37.78 (15.03) | 49.28 (34.80) | 45.75 (32.97) | 70.98 (65.08) | 74.52 (56.98) | 49.70 (31.34) | 79.66 (65.73) |
| INS180 (μU/ml) | 9.24 (3.96) | 27.73 (43.25) | 20.06 (16.01) | 35.09 (29.76) | 39.12 (21.32) | 24.00 (19.10) | 50.85 (35.56) |
| HbA1c (%) | 5.41 (0.49) | 5.59 (0.41) | 5.58 (0.52) | 5.76 (0.41) | 5.90 (0.32) | 5.67 (0.44) | 7.58 (1.50) |
| HOMA-β | 192.06 (89.15) | 169.73 (102.79) | 176.81 (99.88) | 163.93 (101.20) | 144.10 (71.90) | 127.38 (59.90) | 90.08 (63.99) |
| HOMA-IS | 0.56 (0.23) | 0.46 (0.33) | 0.58 (0.62) | 0.39 (0.17) | 0.32 (0.20) | 0.49 (0.16) | 0.27 (0.25) |

**Table S3. Characteristics of CGMformer_types with participants from CGMap.**

|  | **Normal** | **Pre_Ia** | **Pre_Ib** | **Pre_IIa** | **Pre_IIb** | **Pre_IIc** | **All** | **Diabetes** |
| --- | --- | --- | --- | --- | --- | --- | --- | --- |
| **Num (n, %)** | 2840 (34.67%) | 1964 (23.98%) | 2734 (33.38%) | 561 (6.85%) | 49 (0.60%) | 43 (0.52%) | 8191 (100.00%) | 92 (1.12%) |
| **Female (n, %)** | 1683 (59.26%) | 944 (48.07%) | 1439 (52.63%) | 230 (41.00%) | 18 (36.73%) | 21 (48.84%) | 4335 (52.92%) | 38 (41.30%) |
| **PreT2D (n, %)** | 116 (4.08%) | 229 (11.66%) | 193 (7.06%) | 117 (20.86%) | 18 (36.73%) | 6 (13.95%) | 679 (8.29%) |  |
| **Clinical Measurements (Mean (std))** | | | | | | | | |
| **age** | 48.5486 (7.3968) | 50.6555 (7.7731) | 49.9301 (8.1149) | 52.5939 (8.2356) | 54.8837 (9.2833) | 52.7442 (9.3490) | 49.8519 (7.9037) | 56.2000 (6.8230) |
| **fpg** | 90.0393 (8.2038) | 94.6754 (9.1692) | 92.4738 (8.4319) | 97.2376 (9.8343) | 104.1462 (10.2406) | 94.5359 (8.4393) | 92.5999 (8.9748) | 135.9011 (23.9663) |
| **bmi** | 25.6879 (3.9466) | 26.4293 (4.2009) | 25.9736 (4.0470) | 27.1061 (4.3475) | 27.3422 (4.3906) | 27.5324 (5.3533) | 26.0774 (4.1018) | 28.7157 (4.0932) |
| **HbA1C** | 5.3181 (0.3573) | 5.4580 (0.4026) | 5.4001 (0.3829) | 5.5937 (0.3887) | 5.7625 (0.4009) | 5.6385 (0.4610) | 5.4107 (0.3908) | 6.4898 (0.8160) |
| **HDL** | 55.1390 (13.6925) | 52.5751 (12.4671) | 53.7210 (13.2255) | 52.0788 (12.4768) | 48.6875 (11.0583) | 51.5967 (11.6125) | 53.7907 (13.1929) | 47.8846 (14.3418) |
| **LDL** | 119.3818 (31.8142) | 120.9990 (30.3777) | 120.6415 (30.7905) | 123.9934 (30.5170) | 115.9935 (37.4804) | 120.3917 (42.5424) | 120.4844 (31.1480) | 113.2262 (37.6621) |
| **TC** | 195.8715 (42.5012) | 197.0463 (36.4968) | 196.8818 (36.2625) | 200.8353 (35.2196) | 194.6469 (47.4321) | 198.2519 (47.2080) | 196.8211 (38.6682) | 192.7714 (48.1429) |
| **TG** | 106.7294 (90.3136) | 118.0294 (67.4483) | 115.1321 (64.3573) | 129.0311 (60.4918) | 152.5031 (123.4669) | 126.9493 (70.6030) | 114.0368 (75.7301) | 165.0192 (84.4682) |
| **AndroidFat** | 0.3669 (0.1071) | 0.3741 (0.1041) | 0.3709 (0.1032) | 0.3854 (0.1046) | 0.3765 (0.1028) | 0.4162 (0.1124) | 0.3717 (0.1050) | 0.4309 (0.0862) |
| **TrunkFat** | 0.3503 (0.0919) | 0.3532 (0.0906) | 0.3519 (0.0893) | 0.3610 (0.0921) | 0.3516 (0.0945) | 0.3877 (0.0959) | 0.3525 (0.0909) | 0.4002 (0.0765) |
| **TotalFat** | 0.3410 (0.0844) | 0.3343 (0.0826) | 0.3368 (0.0826) | 0.3342 (0.0840) | 0.3200 (0.0887) | 0.3567 (0.0804) | 0.3374 (0.0834) | 0.3622 (0.0712) |
| **CV** | 16.7017 (3.9604) | 14.5048 (3.0936) | 15.9776 (3.7117) | 16.0280 (3.8150) | 16.3896 (7.1213) | 30.9518 (12.8527) | 15.9601 (4.0432) | 20.0131 (4.8513) |
| **TIR** | 85.7632 (13.7198) | 96.1068 (2.6875) | 96.1277 (3.3041) | 88.5205 (5.7216) | 64.4874 (21.2521) | 78.5784 (10.1525) | 91.7266 (10.2707) | 75.3911 (22.2408) |
| **Mean** | 83.6434 (5.3642) | 104.6334 (3.6914) | 94.8599 (2.9297) | 116.3426 (4.7184) | 137.5950 (15.9428) | 108.2721 (9.0122) | 95.1118 (11.3792) | 119.0914 (29.3107) |

**Table S4.** Dataset used in our study.

| Dataset | Sample Num | Source |
| --- | --- | --- |
| Nationwide Multicenter | 450 NGTs, 169 IGRs, 345 T2Ds | Original |
| SIBIONICS | 36,719 T2Ds, 22,128 NDs | Original |
| CGMap | 9,410 NDs | *Cell Metabolism. 2023 May 2;35(5):758-69.* |
| Zhao | 100 T2Ds, 12 T1Ds | *Scientific Data. 2023 Jan 19;10(1):35.* |
| Colas | 208 NDs | *PLoS One. 2019 Dec 18;14(12):e0225817.* |
| CITY | 74 T1Ds | *JAMA. 2020 Jun 16;323(23):2388-96.* |
| SENCE | 14 T1Ds | *Diabetes Care 2021;44(2):464–472* |

**Table S5.** Experiment design and usage of external datasets in our study.

| **Task** | | **Description** | **Training or Finetuning dataset** | **Testing or validation dataset** |
| --- | --- | --- | --- | --- |
| Pretrain | | Pretraining CGMformer to capture glucose dynamics from CGM data. 45~60% glucoses are masked and predicted. | Nationwide Multicenter, SIBIONICS | Nationwide Multicenter, Zhao, Colas, CITY SENCE, SIBIONICS |
| Diagnosis | NGT/IGR/T2D | Diagnosis samples as NGT/IGR/T2D from CGM data | Nationwide Multicenter | Nationwide Multicenter (cross-validation), Colas, SIBIONICS |
|  | T1D/T2D | Diagnosis samples as T1D/T2D from CGM data | Zhao | Zhao (cross-validation) |
|  | Complications | Diagnosis if the samples have diabetes complications from CGM data | T2Ds in Zhao | T2Ds in Zhao (cross-validation) |
| CGMformer_C | | Estimate the impairment of glucose regulation from CGM data | Nationwide Multicenter | Nationwide Multicenter, T2Ds in Zhao |
| Subtyping | | Subtyping the non-diabetes with elevated risks to develop diabetes from CGM data | Nationwide Multicenter | Nationwide Multicenter, Colas, CGMap, |
| Postprandial glucose prediction | | Predict postprandial glucose with CGM records, pre-prandial glucose, and dietary records. | T2Ds in Zhao | T2Ds in Zhao (cross-validation) |

**Table S6. Description for CGM derived Metrics.**

| **Metric** | **Short description** |
| --- | --- |
| above_percent (above_140, above_180, above_250, TAR) | Percentage of measured glucose values above target thresholds. |
| below_percent (below_54, below_70, TBR) | Percentage of measured glucose values below target thresholds. |
| in_range_percent (in_range_63_140, in_range_70_180, TIR) | Percentage of measured glucose values in targeted value ranges. |
| HBGI | High Blood Glucose Index (HBGI) $=\frac{1}{n}*\Sigma(10*fpg_{i}^{2})$, where $fbg_{i}=max(0,1.509*({\log\left( G_{i} \right)}^{1.084}-5.381)$, $BG_{i}$ the $i$th Glucose measurement for a subject, and n is the total number of measurements  for that subject. |
| LBGI | Low Blood Glucose Index (LBGI) $=\frac{1}{n}*\Sigma(10*fpg_{i}^{2})$, where $fbg_{i}=min(0,1.509*({\log\left( G_{i} \right)}^{1.084}-5.381)$, $G_{i}$ the $i$th Glucose measurement for a subject, and n is the total number of measurements  for that subject. |
| ADRR | Average daily risk range, the average sum of HBGI corresponding to the highest glucose value and LBGI corresponding to the lowest glucose value for each day, with the average taken over the daily sums. |
| COGI | Continuous Glucose Monitoring Index (COGI) |
| CONGA | Continuous Overall Net Glycemic Action (CONGA) is the standard deviation of the difference between glucose values that are exactly n hours apart. CONGA is computed by taking the standard deviation of differences in measurements separated  by n hours. |
| CV | Coefficient of Variation (CV) of glucose levels. |
| CV_Measures (CV_Measures_Mean, CV_Measures_SD) | Coefficient of Variation subtypes. CV_Measures_Mean is calculated by first taking the coefficient of variation of each day’s glucose measurements, then taking the mean of all the coefficient of variation. CV_Measures_SD is calculated by first taking the coefficient of variation of each day’s glucose measurements, then taking the standard deviation of all the coefficient of variations. |
| eA1C | Estimate A1C. eA1C score is calculated by $(46.7 + mean(G))/28.7$ where $G$ is the vector of Glucose Measurements (mg/dL). |
| GMI | GMI score is calculated by $3:31+(0.02392*mean(G))$ where $G$ is the vector of Glucose  Measurements (mg/dL). |
| GRADE | Glycemic Risk Assessment Diabetes Equation (GRADE) score is calculated by$\frac{1}{n}*\Sigma\left[ 425*\left( \log\left( \log\left( \frac{G_{i}}{18} \right) \right)+0.16 \right)^{2} \right]$, where $G_{i}$ is the ith  Glucose measurement and n is the total number of measurements |
| grade_eugly, grade_ hyper, grade_hypo | Percentage of GRADE score attributable to target range, hyperglycemia, and hypoglycemia |
| GRI | Glycemia Risk Index (GRI) $=(3.0VLow) + (2.4Low) + (1.6VHigh) + (0.8High)$, where VLow, Low, VHigh, and High correspond to the percent of glucose values in the ranges <54 mg/dL, 54-70 mg/dL, >250 mg/dL, and 180-250 mg/dL respectively. |
| GVP | Glucose Variability Percentage (GVP) is calculated by dividing the total length of the line of the glucose trace by the length of a perfectly flat trace. |
| hyper_index | Hyperglycemia Index is calculated by $\frac{n}{c}*\Sigma\left[ hyperBG_{j}-ULTR \right)^{a}]$, Here n is the total number of Glucose measurements (excluding NA values), $hyperBG_{j}$ is the jth Glucose measurement above the ULTR cutoff, a is an exponent, and c is a scaling factor. |
| hypo_index | Hypoglycemia Index is calculated by $\frac{n}{d}*\Sigma\left[ LLTR-hypoBG_{j} \right)^{b}]$, Here n is the total number of Glucose measurements (excluding NA values), $hypoBG_{j}$ is the jth Glucose measurement below the LLTR cutoff, b is an exponent, and d is a scaling factor. |
| IGC | Index of Glycemic Control (IGC) is calculated by taking the sum of the Hyperglycemia Index and the Hypoglycemia index. |
| IQR | The distance between the 25th percentile and the 25th percentile of the glucose values. |
| J-Index | J-Index score is calculated by $0.001*\left[ mean\left( G \right)+sd\left( BG \right) \right]^{2}$ where $G$ is the list of Glucose Measurements. |
| MAD | Median Absolute Deviation (MAD) is calculated by taking the median of the difference of the glucose readings from their median and multiplying it by a scaling factor $1.4826*median(\vert gl-median(gl)\vert)$, where gl is the list of Blood Glucose measurements. |
| MAG | Mean Absolute Glucose (MAG) is calculated as $\frac{\left\vert\Delta G \right\vert}{\left\vert\Delta t \right\vert}$ where $\vert\Delta G\vert$ is the sum of the absolute change in glucose calculated for each interval as specified by n, default n= 60 for hourly change in blood glucose. |
| MAGE | Mean Amplitude of Glycemic Excursions |
| Mean | Mean glucose level |
| Median | Median glucose level |
| MODD | Mean difference between glucose values obtained at the  same time of day (MODD) |
| M_value | M-value is computed by averaging the transformed glucose values, where each transformed value is equal to $\vert10*\log\left( \frac{\mathrm{glucose}}{r} \right)\left. \right\vert^{3}$, where r is the specified reference value. |
| Quantiles (Min., 1^st^, 3^rd^, Max.) | Minimum, 1^st^ quantile, 3^rd^ quantile, maximum of the input glucose measurements. |
| Range | The distance between minimum and maximum glucose values. |
| SD | Standard Deviation (CV) of glucose levels. |
| SD_measurements (SDw, SDhhmm, SDwsh, SDdm, SDb, SDbdm, SD. Roc) | SD subtype values, including vertical within days (SDw), between time points (SDhhmm), within series (SDwsh), horizontal sd (SDdm), between days with in timepoints (SDb), between days within timepoints and corrected for changes in daily means (SD bdm), SD of the rate of change values (SD. Roc) |

**Table S7. Hyperparameter in CGMformer pretraining and finetuning.**

| **Hyperparameter** | **Value** |
| --- | --- |
| *Pretraining* | |
| Total Number of Parameters | 881,674 |
| Number of Transformer Layers | 4 layers |
| Transformer Layer Embedding Size | 128 |
| Transformer Layer Hidden Dimension | 512 |
| Number of Transformer Heads | 8 heads |
| Initializer_range | 0.02 |
| Layer_norm_eps | 1e-12 |
| Attention_probs_dropout_prob | 0.02 |
| Hidden_dropout_prob | 0.02 |
| Weight_decay | 0.001 |
| Max_lr | 4e-4 |
| lr_schedule_fn | linear |
| optimizer | adamw |
| Transformer Layer Activation Function | gelu |
| Transformer Layer Normalization | LayerNorm |
| Pre-trained CGM sequence length | 288 |
| Total Training Epochs | 3000 epochs |
| Warmup Epochs | 2000 epoch |
| GPU type | NVIDIA 3080Ti-12GB |
| Number of Nodes | 1 machine |
| Total Number of GPUs | 2 GPUs |
| Per GPU Batch Size | 48 |
| Token Embedding MLP Layer Dimensions (Transformer) | 512,128 |
| MLP Layer Activation Function | gelu |
| MLP Layer Normalization | LayerNorm |
| Total Training Time | 3.5h |
| *Fine-tune for T1D/T2D diagnosis and complication diagnosis in Zhao’s dataset* | |
| Max_lr | 4e-4 |
| Frozen layers | 0 |
| Num_gpus | 1 |
| Num_proc | 16 |
| Batch_size | 48 |
| lr_schedule_fn | cosine |
| epochs | 20 |
| warmup_steps | 100 |
| optimizer | adamw |
| Prediction Decoder MLP Layer Dimensions (Transformer) | 512,128,2 |
| *Fine-tune for NGT/IGR/T2D diagnosis in nationwide multi-center dataset* | |
| Max_lr | 4.1e-4 |
| Frozen layers | 0 |
| Num_gpus | 1 |
| Num_proc | 16 |
| Batch_size | 12 |
| lr_schedule_fn | cosine |
| epochs | 20 |
| warmup_steps | 1300 |
| optimizer | adamw |
| Prediction Decoder MLP Layer Dimensions (Transformer) | 512,128,3 |

**Supplementary figures**

**
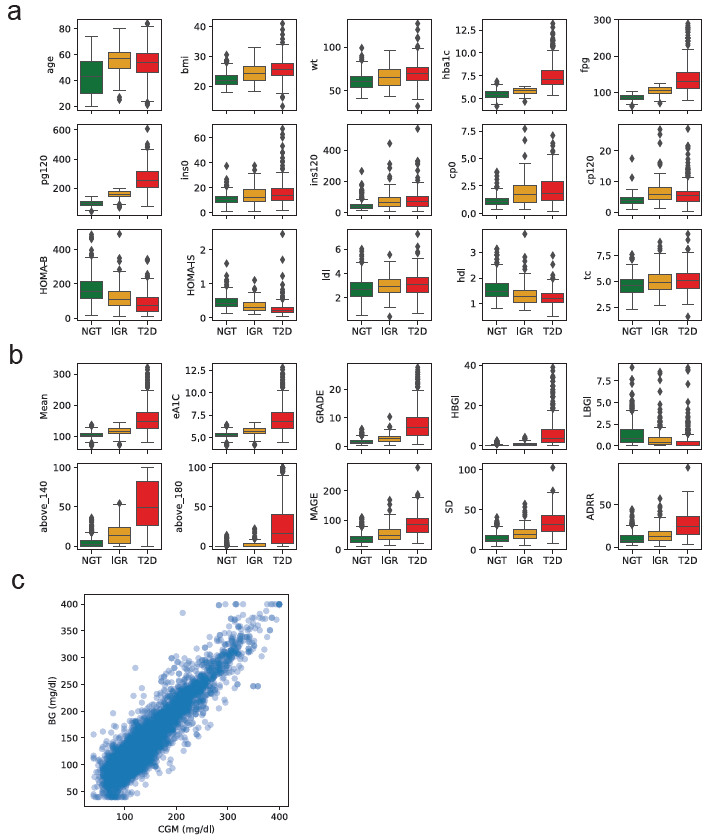
**

**Fig S1. Nationwide multi-center CGM study.** a. Clinical measurements in nationwide multi-center CGM study. wt: Weight(kg). b. CGM derived metrics in nationwide multi-center CGM study. c. Scatter for measurements from CGM and fingerstick glucose measurement (BG).


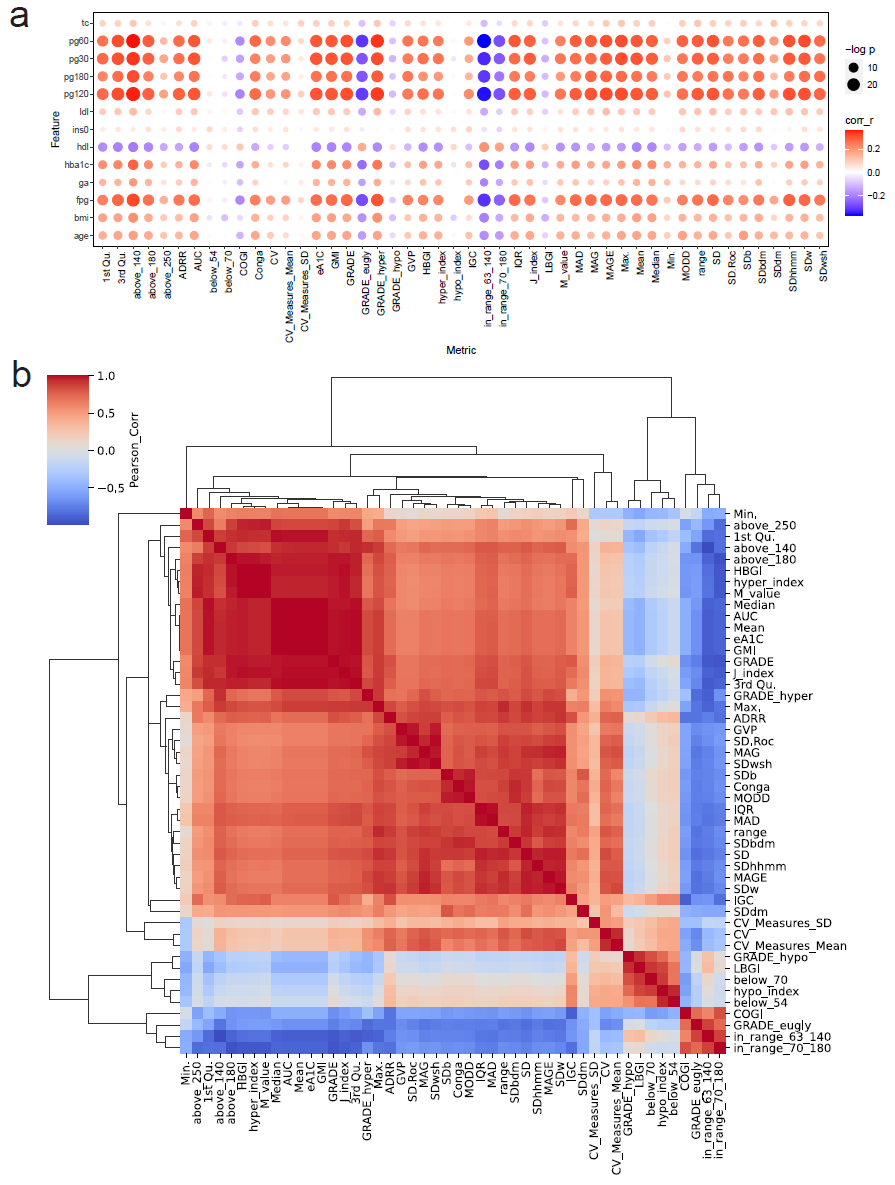


**Fig S2. CGM data collected from Nationwide multi-center CGM study.** a. Correlation between CGM-derived metrics and clinical measurements. Descriptions for the CGM-derived metrics are listed in **Table S4**. b. Correlation analysis of CGM-derived metrics.

**
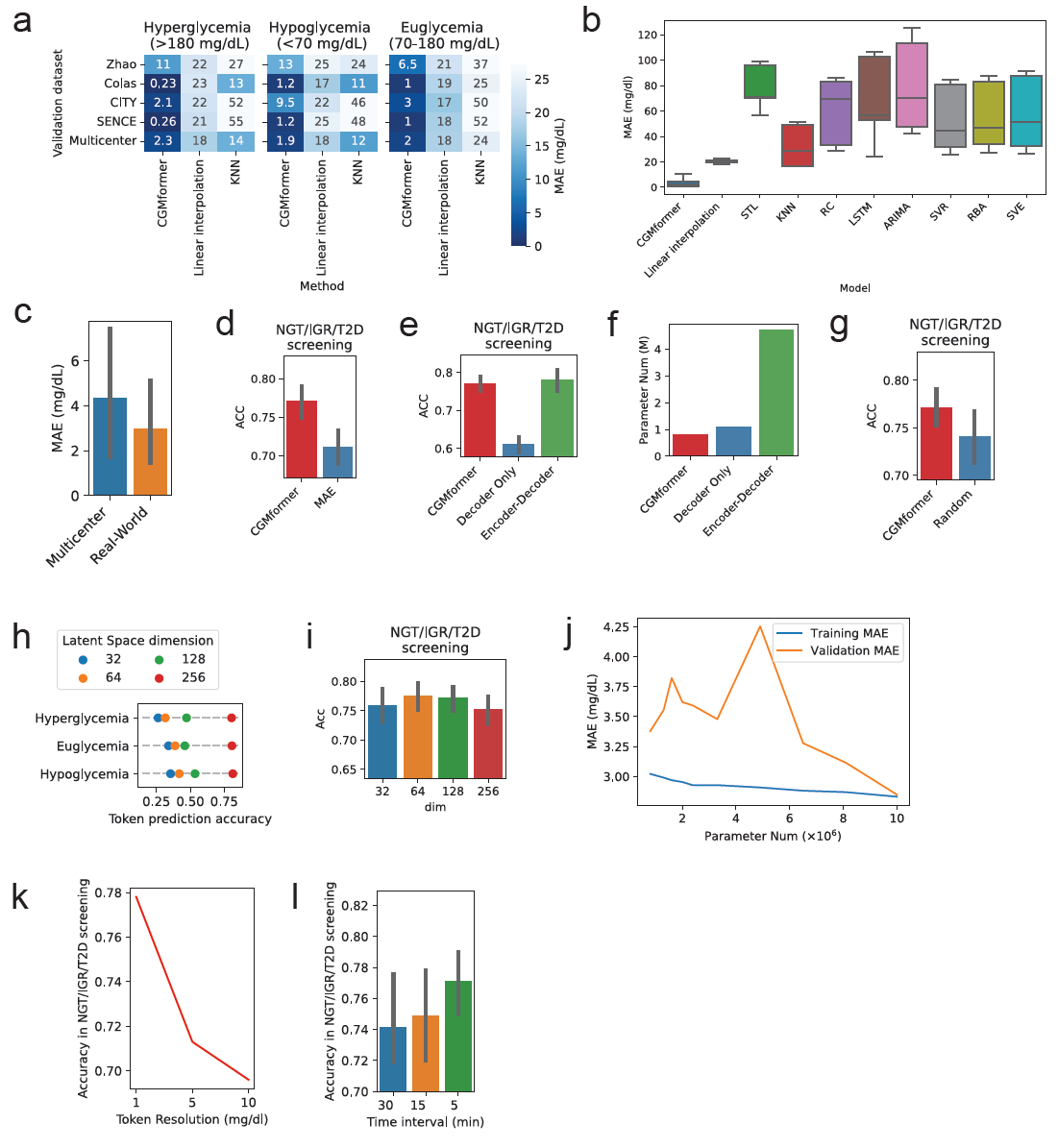
**

**Fig S3. Ablation study for model hyperparameters.** a. MAE for token prediction (left: normal glycemia, center: hyperglycemia, right hypoglycemia) after pretraining comparing with linear interpolation and KNN. b. Accuracy for token prediction after pretraining comparing with other baseline methods. c. Accuracy for token prediction comparing model pretrained on Nationwide Multi-center CGM study and National Real-world CGM data. d. Accuracy for label prediction (NGT/IGR/T2D diagnosis) comparing with model with MAE loss. e. Accuracy for label prediction (NGT/IGR/T2D diagnosis) with different model architecture. f. Parameter num different model architecture. g. Accuracy for label prediction (NGT/IGR/T2D screening) with different masking strategies. h. Accuracy for token prediction for model with different latent space dimension. i. Accuracy for label prediction (NGT/IGR/T2D screening) for model with different latent space dimension. j. MAE for token prediction for model with increasing parameters. k. Accuracy for label prediction (NGT/IGR/T2D screening) for model with different tokenize resolution. l. Accuracy for label prediction (NGT/IGR/T2D screening) for input with different time interval.

**
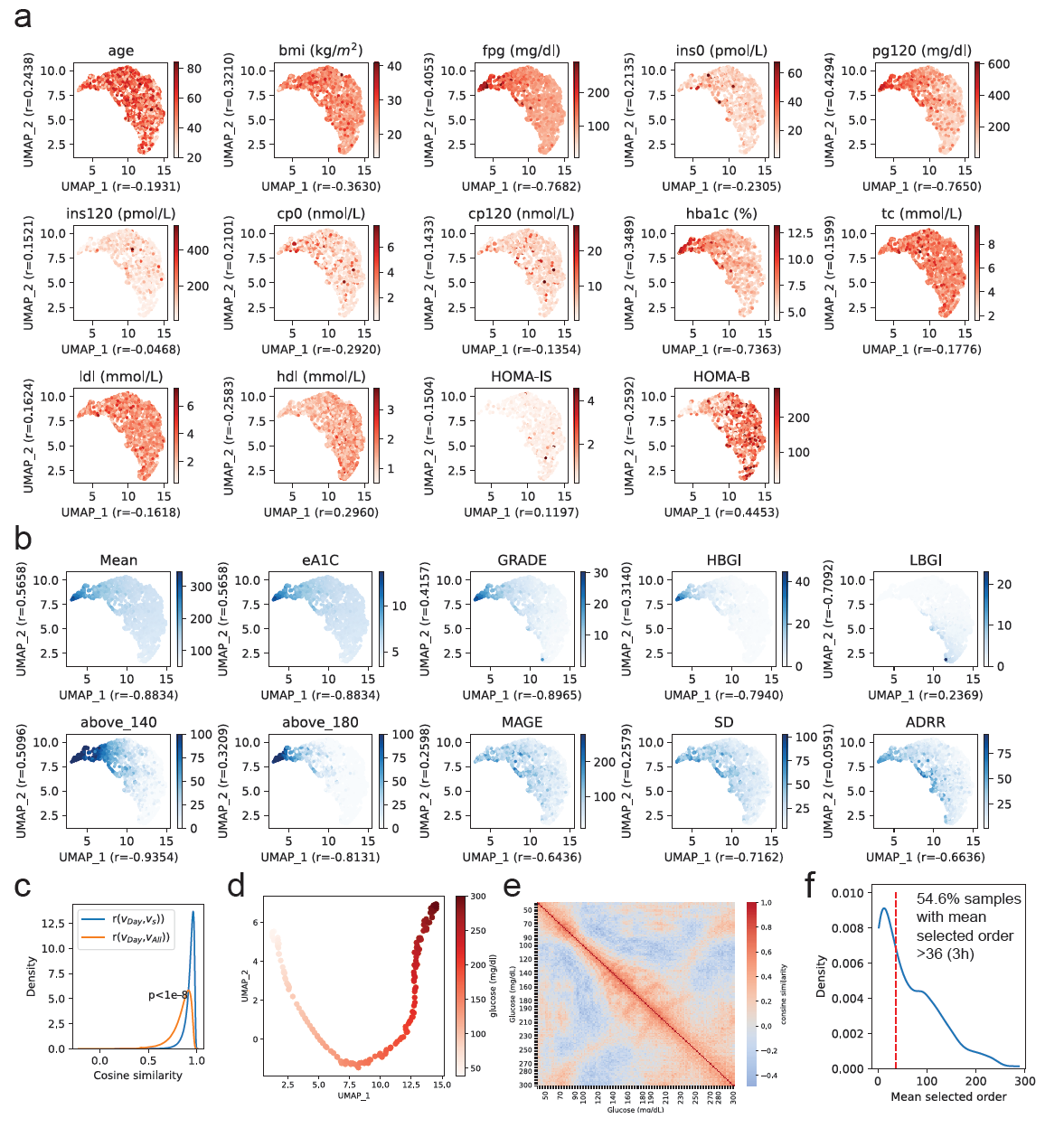
**

**Fig S4. UMAP visualization for individual clinical measurements and CGM derived metrics.** Pretrained CGMformer individual embeddings align well with individual clinical measurements (a) as well as CGM-derived measurements (b). CGM-derived metrics in (b) includes: Mean: Mean glucose level, eA1C: estimated A1C, GRADE: Glycemic Risk Assessment Diabetes Equation score, HBGI: High Blood Glucose Index, LBGI: Low Blood Glucose Index, above_140: percentage of measured glucose values above threshold (140mg/dl), above_180: percentage of measured glucose values above threshold (180mg/dl), MAGE: Mean Amplitude of Glycemic Excursions, SD: standard deviation, ADRR: average daily risk range. Detailed descriptions for these metrics refer to **Table S4**. c Distribution for cosine similarity of singly-day embeddings intra- or inter- samples. d. UMAP for glucose level tokens embedding after pretraining. e. Heatmap for glucose level tokens embedding correlation after pretraining. f. Distribution for mean selected order in AIC.

**
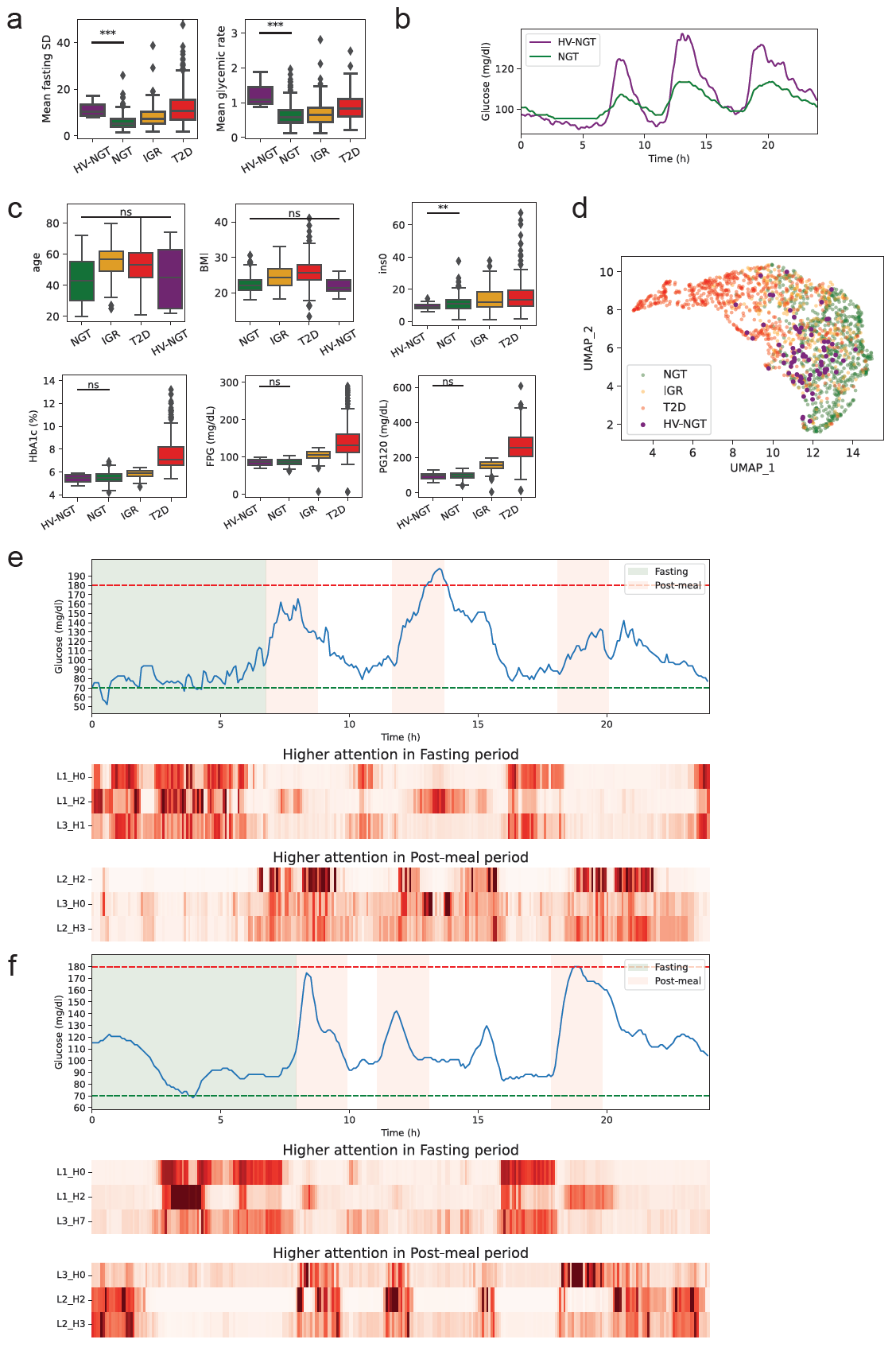
**

**Fig S5.** a. Mean fasting SD and mean glycemic rate in HV-NGT and NGT. b. Median CGM profile form HV-NGT and NGT.c. Comparison of age, BMI, fasting insulin, HbA1c, FPG, and PG120 among individual from NGT, IGR, T2D, and HV-NGT. d. UMAP visualizations for embedding vectors of HV-NGTs. e. CGM profile and corresponding attention weights from another day recorded by Shanghai_NGT_A183. f. Another example from HV-NGT show the attention weights can learn the dynamics during fasting phase and post-meal phase.

**
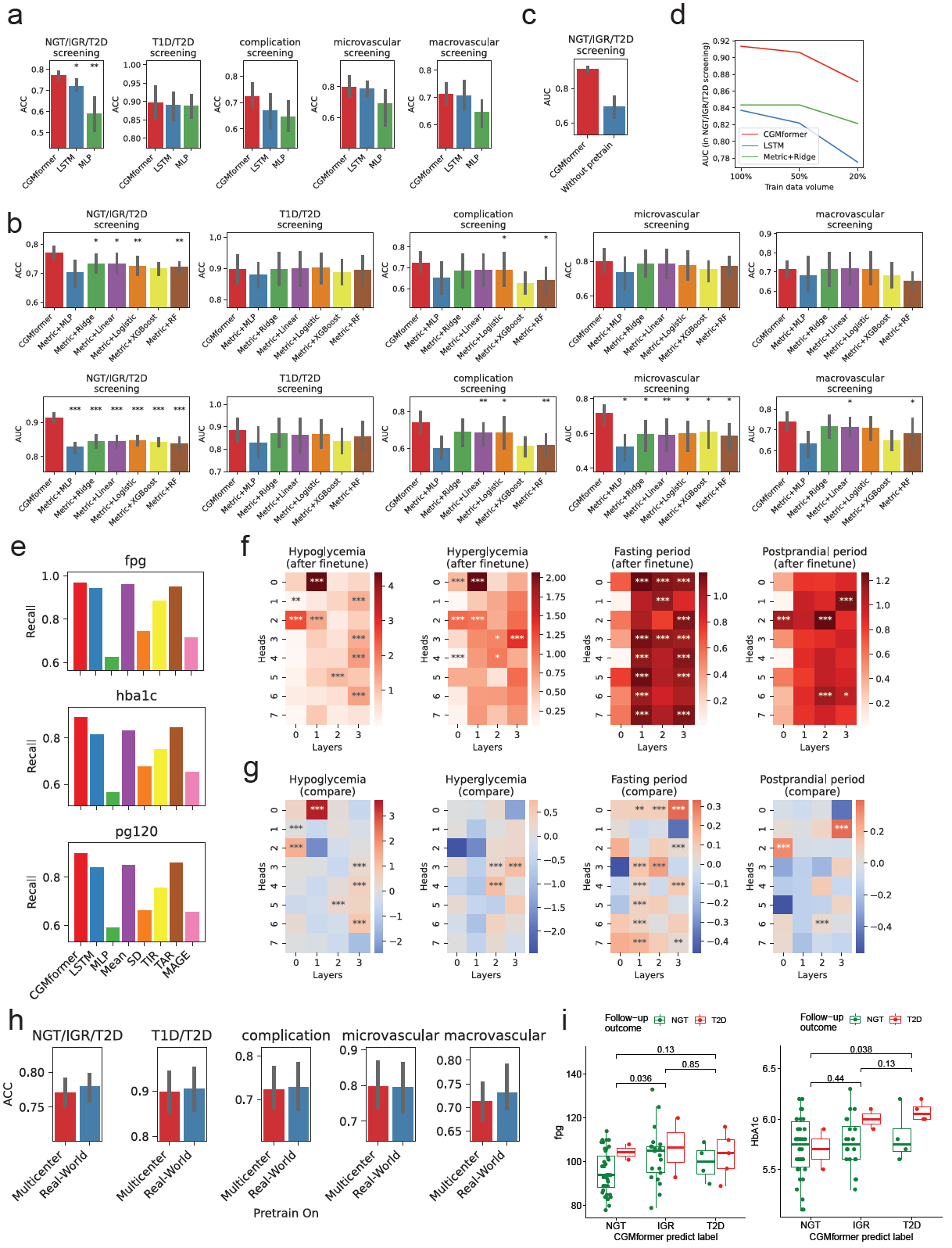
**

**Fig S6.** a. ACC for screening NGT/IGR/T2D, T1D/T2D, complication, microvascular, and macrovascular, comparing with machine learning methods including MLP and LSTM. b. Accuracy and AUC for assisting diagnosis NGT/IGR/T2D, T1D/T2D, complication, microvascular, and macrovascular, comparing with combining CGM derived metrics with feature extracting methods including Ridge regression classifier, MLP classifier, and SGD classifier. c. AUROC for screening NGT/IGR/T2D with model with or without pretraining. d. AUROC for screening NGT/IGR/T2D with decreasing finetuning training data. e. Recall score in predicting T2D from different clinical critical, comparing with baseline methods, including machine learning methods and diagnosis from CGM derived metrics. Clinical critical for different measurements are as follows: FPG$\geq$7mmol/L; HbA1c$\geq$6.5%; PG120$\geq$11.1mmol/L. f. Heatmap for attention weights in different glucose level and dynamic phase after finetuning. g. Comparison for attention weights in different glucose level and dynamic phase after finetuning with pretraining. h. ACC for screening NGT/IGR/T2D, T1D/T2D, complication, microvascular, and macrovascular, comparing model pretrain on the Nationwide Multicenter CGM study or National Real-world CGM data, i. Comparison of FPG (left) and HbA1c (right) from samples grouped with finetuned CGMformer predicted results, hued by follow-up outcome.


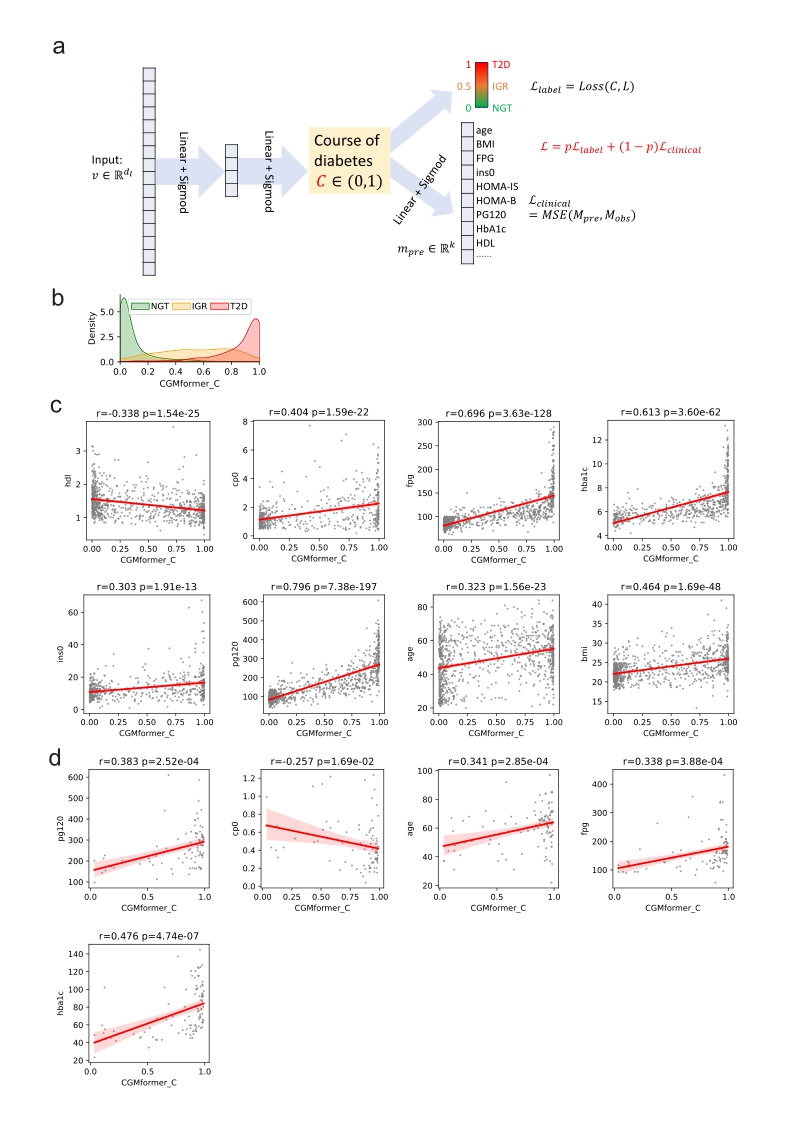


**Fig. S7.** a. Schematic for calculating CGMformer_C from embedding vector encoded by CGMformer. The embedding vectors are encoded through 2-linear layers into 1-dimensional indexes, and then decoded with multi-task regression loss-function to optimize model and obtain CGMformer_C. b. KDE plot estimates the distribution of CGMformer_C from NGT/IGR/T2D. cd. Scatter plot for CGMformer_C with clinical measurements from nationwide multi-center dataset (c) and Zhao’s dataset (d).


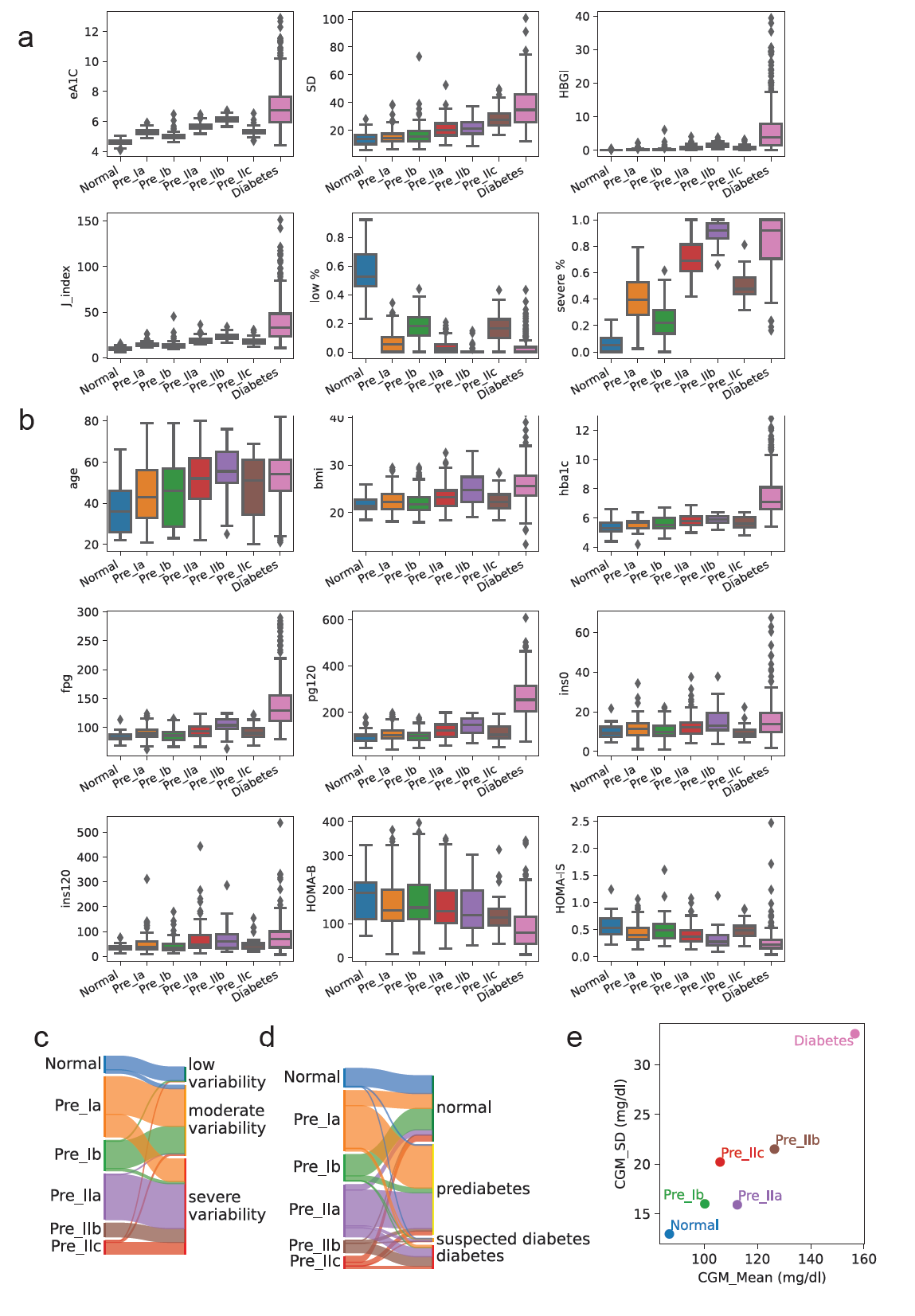


**Fig S8.** a. CGM derived metrics for individuals from each subtype. HBGI: high blood glucose index. low%: fraction of time with low glucose variability, calculated from glucotype; severe %: fraction of time with severe glucose variability, calculated from glucotype. b. Clinical measurements for individuals from each subtype. cd. Comparison of CGMformer_type with Glucotype (c) and CGM FG type (d). e. Mean glucose level (CGM_Mean) and glucose variability (CGM_SD) for subtyping based on embeddings generated from model pretrained on National Real-world CGM data.


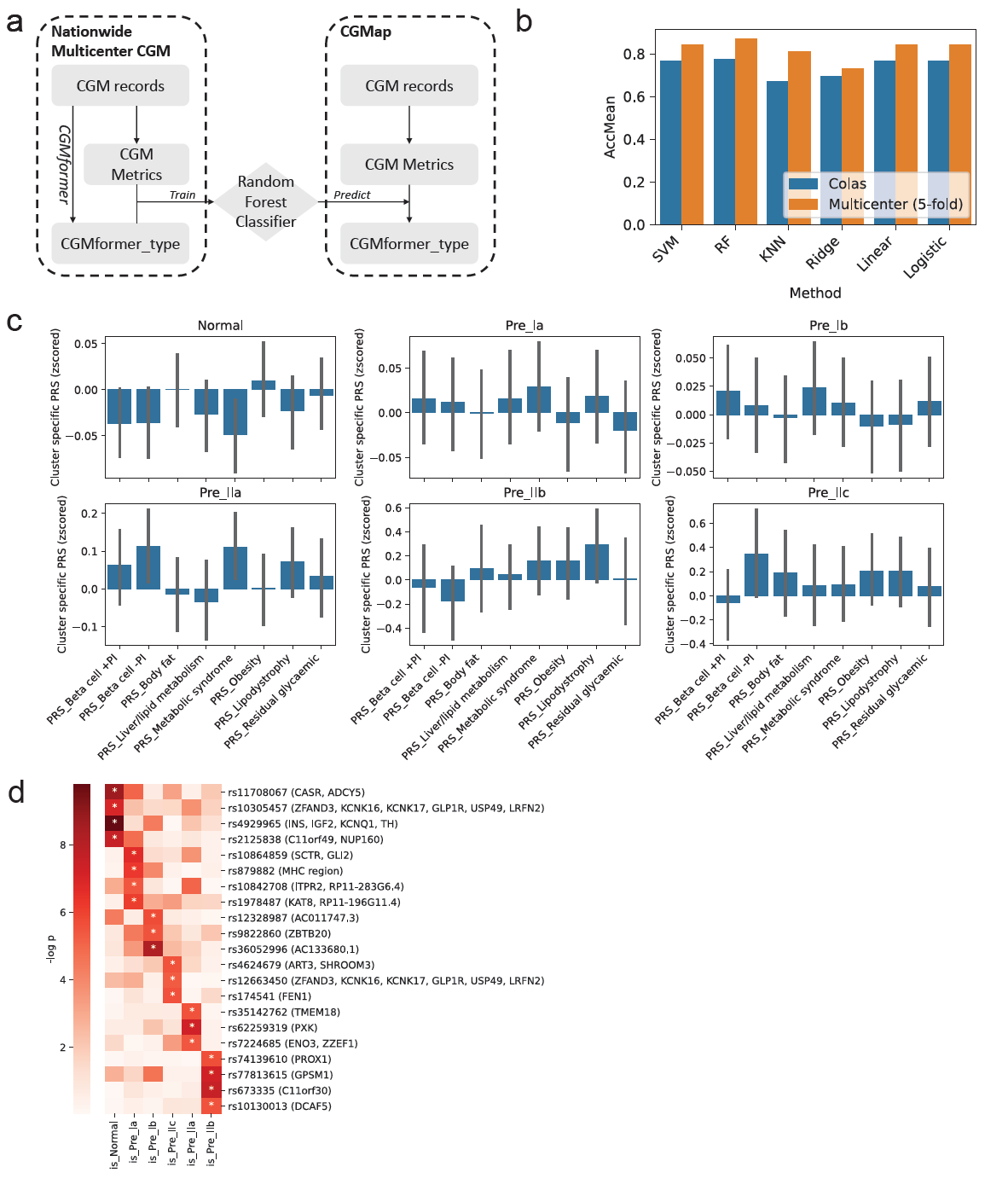


**Fig S9.** a. Schematic for annotating samples in CGMap with CGMformer_type. b. ACC for different classifiers predicting CGMformer_type from CGM metrics. c. Bos plot for cluster specific PRS of diabetes for samples with each CGMformer_type from CGMap. d. SNPs and corresponding gene regions which significantly correlated with CGMformer_type.


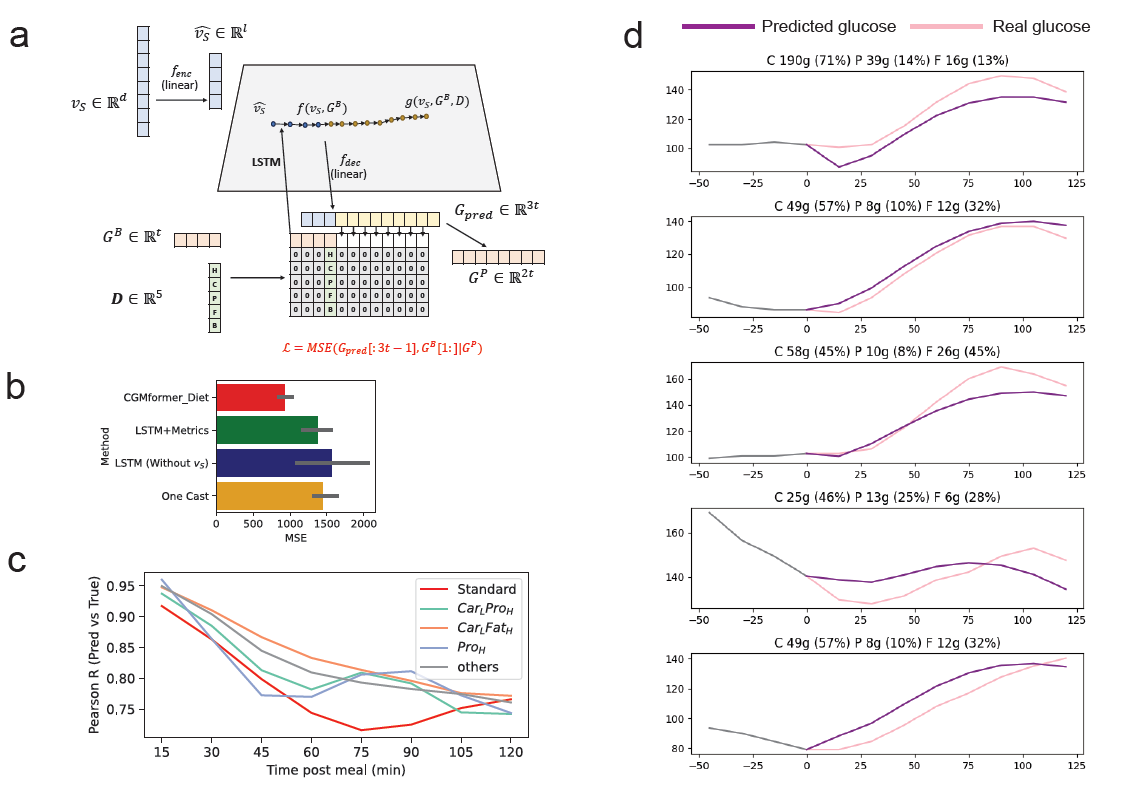


**Fig S10.** a. Detailed schematic for CGMformer_Diet. b. MSE for CGMformer_Diet predicted post-meal glucose with baseline methods. c. Pearson correlation coefficient for CGMformer_Diet predicted post-meal glucose with real post-meal glucose, grouped by different energy supply ratio of meal intake. d. Comparison of predicted post-meal glucose with real post-meal glucose of several meals from one individual validates the results for dietary perturbation based on CGMformer_Diet.
